# Intracellular Delivery of Proteins for Live Cell Imaging

**DOI:** 10.1016/j.jconrel.2025.113651

**Published:** 2025-03-20

**Authors:** Ban-Seok Jeong, Hwanhee Chris Kim, Catherine M. Sniezek, Stephanie Berger, Justin M. Kollman, David Baker, Joshua C. Vaughan, Xiaohu Gao

**Affiliations:** 1. Department of Bioengineering, University of Washington; 2. Department of Chemistry, University of Washington; 3. Institute for Protein Design, University of Washington; 4. Department of Biochemistry, University of Washington; 5. Howard Hughes Medical Institute, University of Washington; 6. Department of Neurobiology and Biophysics, University of Washington

**Keywords:** cytosolic delivery, small molecule, protein, antibody, live-cell imaging, drug discovery

## Abstract

The majority of cellular functions are regulated by intracellular proteins, and regulating their interactions can unlock fundamental insights in biology and open new avenues for drug discovery. Because the vast majority of intracellular targets remain undruggable, there is significant current interest in developing protein-based agents especially monoclonal antibodies due to their specificity, availability, and established screening/engineering methods. However, efficient delivery of proteins into the cytoplasm has been a major challenge in biological engineering and drug discovery. We previously reported a platform technology based on a Coomassie blue-cholesterol conjugate (CB-tag) capable of delivering small proteins directly into the cytoplasm. Here, we report a new generation of CB-tag that can bring proteins with a wide size range into the cytoplasm, bypassing endosomal sequestration. Remarkably, intracellular targets with distinct structures were visualized. Overall, the new CB-tag demonstrated a robust ability in protein delivery with broad applications ranging from live-cell immunofluorescence to protein-based therapeutic development.

## Graphical Abstract

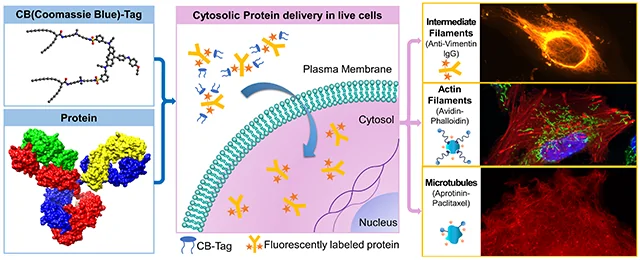

## INTRODUCTION

Intracellular delivery of functional proteins to live cells holds tremendous potential for the development of therapeutics and diagnostics, such as understanding protein-protein interactions, replacing defective enzymes, modulating signaling pathways, and promoting targeted protein degradation.^1^ Because of the large binding interfaces, proteins can interact with a wide range of biological targets, many of which have been considered “undruggable” by traditional small-molecule drugs. This unique advantage enables them to distinguish subtle differences in targets, such as post-translational modifications, point mutations, quaternary structures, and splice variants with high specificity and affinity.^2–5^ Hence, various protein-based affinity reagents such as antibodies, antibody fragments, nanobodies, affibodies, monobodies, DARPins, and de novo-designed minibinders have been developed for imaging and therapeutic applications.^6–13^

Among them, antibodies play a prominent role because of the broad availability of *in vitro* and *in vivo* validated antibodies.^14^ However, their use has been largely limited to the extracellular space including membrane receptors and secreted antigens due to the impermeability of proteins through the membrane of live cells.^15^ This limitation has resulted in intracellular therapeutic targets, which comprise approximately 70% of all proteins encoded by the human genome,^16^ underexplored.

To expand the scope of antibodies to treat intracellular targets and perform live-cell immunofluorescence imaging, efficient and robust cytosolic protein delivery is crucial. In this context, a wide range of delivery technologies have been developed based on cell-penetrating peptides,^17–20^ inorganic nanomaterials,^21, 22^ polymers,^23–33^ and lipids,^34–36^ but the delivery efficiency is often hampered by the endocytic cell entry pathway where protein cargos are degraded inside endosomes and lysosomes.^37–40^ To tackle this bottleneck, recent strategies focused on direct translocation of proteins across the plasma membrane to avoid the difficulties of endosomal escape and to deliver proteins into hard-to-transfect cells that have low endocytic activities. Several methods rely on transient membrane disruption using physical techniques such as microinjection, electroporation, sonoporation, photoporation, and microfluidics.^41–43^ These methods bring down the membrane barrier between the extracellular and intracellular spaces for cargo protein entry but require high concentrations of the cargo protein and often result in cell injury and reduced viability.^44^ Alternatively, chemical approaches based on the complexes of protein and cationic gold nanoparticles,^45–48^ polymers,^49–51^ phase separating peptides,^52–55^ or lipids^56^ have also been developed to translocate proteins directly into the cytosol. These complexes can quickly enter the cytosol after getting in contact with the cell membrane.^57, 58^

In this context, our group recently developed a unique biologic cytosolic delivery technology by non-covalently tagging proteins with cholesterols.^59^ The non-covalent association was achieved by modifying cholesterol molecules with CB, a protein-binding dye. When mixed with protein molecules, the CB end binds to proteins while the cholesterol end faces outward. Biomolecules non-covalently tagged by cholesterols were believed to enter cells through an initial membrane fusion followed by biomolecule-tag dissociation. The hydrophobic tag molecules are largely confined in the hydrophobic lipid bilayer while the hydrophilic biomolecule can quickly slip into the cytosol, bypassing the endocytosis. Although the exact mechanism of cell entry is not entirely clear, remarkable delivery efficiency has been observed for both small RNAs and proteins. A key limitation, however, is the protein size. While small proteins such as aprotinin (6.5 kDa) and lysozyme (15 kDa) showed high cytosolic delivery efficiency manifested by bright, diffuse, cytosolic fluorescence, large proteins such as BSA (66 kDa) and IgG (150 kDa) exhibited large puncta with low-level diffuse fluorescence, indicating significant endosome entrapment. As a result, although the concept of CB-tag is appealing, its application scope is limited to small protein binders whose current availability is no match to that of full-size antibodies. Here, we report the design of the second-generation CB-tag in which the bulky cholesterol was replaced by branched alkyl chains (Figure 1a). This new and structurally simpler tag can deliver large proteins including antibodies into the cytosol at high efficiency, substantially expanding the application of CB-tags.

## MATERIALS & METHODS

### (1) Materials and Instruments.

Triethylamine (TEA), Diisopropylethylamine (DIPEA), Triton X-100 (TX-100), Cytochalasin D, Methyl-β-cyclodextrin, Chlorpromazine hydrochloride, 5-(N-ethyl-N-isopropyl)amiloride, Sodium azide, Aprotinin, Bovine serum albumin (BSA), Lysozyme, Cytochrome-C (Cyt-C), Transferrin, Myoglobin, β-Lactoglobulin, Horseradish Peroxidase, Ovalbumin, and Avidin were purchased from Sigma-Aldrich (St. Louis and Burlington, MA). Alexa fluor 488, 555, 568 – NHS esters, Protein G-AF488, Streptavidin-AF555, Goat anti-Rabbit 2° IgG-AF488, Goat anti-Rabbit 2° IgG-AF488 plus, F(ab’)2-Goat anti-Mouse IgG-AF488, Goat anti-Rabbit 2° IgG-AF555, Goat anti-Rabbit 2° IgG-AF568, Vimentin monoclonal antibody (V9), Vimentin monoclonal antibody (V9)-AF488, Vimentin monoclonal antibody (V9)-AF555, Beta-actin monoclonal antibody (15G5A11/E2)-AF555, Alpha tubulin monoclonal antibody (TU-01)-AF555, Beta tubulin monoclonal antibody (BT7R)-AF555, SM(PEG)_2_, SM(PEG)_12_, NHS-PEG_12_-Biotin, Hoechst 33342, and MitoTracker Green FM were purchased from ThermoFisher (Waltham, MA). Phalloidin-lysine was purchased from AAT Bioquest (city, state). 7-O-(Amino-PEG_4_)-paclitaxel was purchased from BroadPharm (San Diego, CA). Phosphate-buffered saline (PBS), Dulbecco’s Modified Eagle’s Medium (DMEM), Opti-MEM, fetal bovine serum (FBS), and penicillin/streptomycin were obtained from Gibco (Waltham, MA). CellTiter-Blue cell viability assay kit was purchased from Promega. μ-Slide 4-well glass bottom chambered coverslips were purchased from Ibidi (Fitchburg, WI). NMR spectra were obtained on Bruker AVANCE I 300 MHz and AVANCE III 500 MHz spectrometers and the chemical shifts are reported in parts per million (ppm). Electron spray ionization (ESI)-mass spectra (MS) were measured on a Bruker EsquireLC ion trap mass spectrometer.

### (2) Protein fluorescence Labeling.

Proteins were dissolved in pH 9.5 Borate buffer saline (10 mM) at 5 mg/mL, and fluorescent dye (AF488, AF555, or AF568) stock solution in DMSO (10 mg/ml) was added into the protein solution and quickly mixed by pipetting. The molar ratio of dye to protein was typically 10:1. The reaction mixture was placed on a rocker for overnight incubation in the dark at room temperature. The excessive unconjugated dyes were removed by desalting spin columns while the buffer was exchanged for phosphate buffer (pH 7.4, 10 mM Phosphate, 0 mM NaCl). The obtained protein solution was diluted to 10 – 50μM and stored at 4 °C in the dark. The degree of labeling of Alexa Fluor 488, 555, or 568 to proteins was calculated from UV-Vis absorption spectra of the labeled proteins, according to the manufacturer’s protocol. For antibodies supplied with stabilizing proteins such as BSA, protein A/G spin column (Thermo Scientific) was used following the manufacturer’s protocol. For proteins pre-labeled with fluorescent dyes, a simple buffer exchange to phosphate buffer (10 mM phosphate, 0 mM NaCl) was performed first.

### (3) Conjugating Paclitaxel or Phalloidin to Aprotinin-AF555.

Aprotinin-AF555 (40 μM) was treated with 5 mM TCEP in PBS for 30 min at RT, followed by purification to remove unreacted TCEP and buffer exchange to phosphate buffer (10 mM phosphate, 0 mM NaCl) using desalting spin columns. Paclitaxel- or Phalloidin-maleimide conjugates (1 mM in DMSO) at a molar ratio of 4:1 (PTX or Phalloidin conjugate:Aprotinin) was added to the protein solution and quickly mixed by pipetting. The mixture was stirred for 60 min at RT in the dark.

### (4) Cell culture.

HeLa (human cervical carcinoma) and COS-7 (African green monkey kidney fibroblast cells) were cultured in DMEM supplemented with 10% FBS and 1% penicillin and streptomycin at 37 °C and 5% CO_2_.

### (5) Protein tagging by the CB-tag.

CB-tag was dissolved in ethanol/water (4:1, v/v) to a concentration of 1.6 mM and stored at -20°C as a stock. The concentration of the protein solutions was adjusted between 1 and 40 μM (depending on the available concentration of commercially obtained proteins) in 5 mM phosphate buffer containing 0.015% (w/v) TX-100. The CB-tag solution was added to the protein solution and quickly mixed by pipetting. Typically, the mixture was incubated for 30 min at RT, followed by further dilution with Opti-MEM to a final protein concentration of 50 nM before adding to cells. For (strept)avidin and IgG, incubation times of 5 min and 15 min, respectively, were sufficient.

### (6) Intracellular protein delivery.

Cells were seeded into 24-well plates at a seeding density of 5.0 × 10^4^ cells per well one day before the experiments. For confocal microscopy, cells were seeded onto μ-Slide 4 well glass coverslips at a seeding density of 6.3 × 10^4^ cells per well. The experiments were conducted at about 80% confluency to avoid cell senescence. The cell culture medium was gently aspirated, and the cell monolayer was washed once with pre-warmed Opti-MEM. After the removal of Opti-MEM, the tagged protein was added to cells (*e.g.*, 400 μl per well for 24-well plates) and incubated for 2 h. Subsequently, the cells were washed with Opti-MEM, counterstained with Hoechst 33342 (100 ng/mL, 30 min), and imaged immediately.

### (7) Cell viability.

Cell viability was determined using Alamar Blue either immediately after or 24 h post protein delivery. 80 μl of CellTiter-Blue reagent (Promega) was added and the culture was incubated for 1 hour before being assessed on a microplate reader (Tecan) at excitation/emission wavelengths of 560/590 nm. Viability was calculated according to the manufacturer’s protocol.

### (8) Endocytosis inhibition assay.

HeLa cells were pre-treated with DMEM containing cytochalasin D (CytD, 10 μM), 5-(N-ethyl-N-isopropyl)amiloride (EIPA, 50 μM), Methyl-β-cyclodextrin (MβCD, 10 mM), chlorpromazine (CPZ, 15 μM), or sodium azide (NaN_3_, 100 mM) for 1 hour in a cell incubator. Untreated cells were used as control. The cells were washed once with Opti-MEM, and the media were replaced by Opti-MEM containing tagged Aprotinin-AF555. After 2-h incubation, cells were washed once with Opti-MEM and counterstained with Hoechst 33342, and observed immediately by fluorescence microscopy. To study the effect of low temperature (4°C), HeLa cells were incubated at 4 °C for 30 min before protein delivery. The cells were washed once with cold media (Opti-MEM) and treated with a cold solution of tagged protein in Opti-MEM. The cells were incubated at 4 °C for 3 hours before washing with cold media and imaging.

### (9) Microscopy.

For confocal microscopy, a Zeiss LSM 710 confocal laser-scanning microscope fitted with a plan apochromat objective (100× or 60x) was used. Alexa Fluor 488 was excited with a 488 nm argon ion laser, and fluorescence was recorded through a frame scan. Hoechst 33342 was excited with a 405 nm diode laser. Alexa Fluor 555 and 568 were excited by a 561 nm HeNe laser. For epi-fluorescence microscopy, an Olympus IX-71 inverted microscope was used with 40x and 10x objectives.

### (10) Live cell time-lapse imaging.

Time-lapse images were captured every 30 s on an inverted Nikon Eclipse Ti2 fluorescence microscope equipped with a stage-top incubator (humidified with 5 % CO_2_ at 37 °C). Before protein delivery, cells were preincubated with Hoechst 33342 (100 ng/mL, 30 min) and MitoTracker Green FM (100 nM, 30 min). Cells were washed once with pre-warmed Opti-MEM, added with tagged proteins in 400 μl Opti-MEM, and immediately moved into the stage-top incubator and imaged.

## RESULTS AND DISCUSSIONS

### (a) Synthesis of the new CB-tag and optimization of delivery conditions.

The synthetic route is illustrated in Figure 1b. Briefly, the sulfonic acid groups of CB G-250 were activated with phosphorus(V) oxychloride for conjugation with compound 7. Detailed chemical characterizations such as mass spectrometry and ^1^H NMR of the intermediates and final product confirmed the molecular structures and purity of the new tag (Supplementary Figures S1–8). The molecular weight and the isotopic pattern of the tag match the expected values, proving successful synthesis of the second-generation CB-tag (Supplementary Figure S8).

Using the new tag, we first optimized the protein intracellular delivery conditions using bovine serum albumin (BSA) as a model cargo because of its availability and the challenge of efficient delivery with the first-generation CB-tag. The BSA molecules were labeled with Alexa Fluor 488 (AF488) with 4 dyes per protein molecule as estimated by UV-Vis absorption. This degree of labeling can be adjusted depending on protein size (higher for larger proteins). In an optimized buffer that contains a trace amount of TX-100 (0.015%) that helps reduce protein aggregation during tagging and improve delivery efficiency, the fluorescently labeled BSA and CB-tag (molar ratio: 1:10) were incubated for 30 minutes before being diluted in Opti-MEM (final concentrations BSA 50 nM, and CB-tag 500 nM) and added to cells for intracellular delivery (2-h incubation). Note that although the final concentration of TX-100 was very low (1.5x10^−4^%) and non-toxic to cells, it was highly effective in preventing the tagged proteins from aggregation. It has been well documented that TX-100 becomes toxic to cells when its concentration reaches the critical micelle concentration (CMC) in the range of 0.2 mM^60^ (~ 100x higher than the conditions used here). Remarkably, the new CB-tag not only brought fluorescent BSA molecules inside the cytosol at high efficiency (>90% cells showing bright and homogeneous fluorescence without significant punctate fluorescent clusters, Figure 1c), but also achieved it at a BSA concentration of 50 nM, 20-fold lower than previously used conditions (1 μM).^59^ This optimized condition can substantially improve the affordability of protein-based imaging and drug discovery studies, and reduce potential toxicity caused by the CB-tag. Quantitative cell viability studies showed that at a CB-tag concentration of 500 nM, the toxicity was negligible with or without the BSA cargo (50 nM) (Figure 1d).

### (b) Intracellular delivery of proteins of various sizes.

After optimizing the protocol of cytosolic delivery using BSA, proteins with a wide range of molecular weight (M.W., 6.5 - 150.0 kDa) and net charge (pI 4.2 - 10.7) were tested. In our previous report, only small proteins such as aprotinin (M.W. 6.5 kDa) and lysozyme (M.W. 15 kDa) could be efficiently transported into the cytosol. Larger proteins had a greater fraction of proteins trapped in endosomes over delivered cytosolically. In the current work, by changing cholesterol in the original CB-tag to branched alkyl chains, remarkably, large proteins such as BSA (M.W. 66.4 kDa), and full IgG (secondary IgG, M.W. 150 kDa) exhibited strong diffuse fluorescence indicating efficiently delivery to the cell cytosol (Figure 2). Among the 15 proteins tested, it was also interesting to observe that small proteins especially the ones with high PI values (positively charged at neutral pH) also had strong nucleus localization, possibly due to the high negative charges of nucleic acids. This protein nuclear localization also proved that the delivered proteins were not trapped inside endosomes.^61^

### (c) Confirmation of non-endocytic cell entry.

To further confirm the new CB-tag brings proteins inside cells via a non-endocytic pathway, we investigated the cellular entry mechanism by pretreating cells with common endocytosis inhibitors including cytochalasin D (actin microfilament depolymerizer), 5-(N-ethyl-n-isopropyl)amiloride (EIPA, pinocytosis inhibitor), chlorpromazine hydrochloride (clathrin-mediated endocytosis inhibitor), sodium azide (NaN_3_, energy-dependent endocytosis inhibitor), and methyl-β-cyclodextrin (MβCD, cholesterol depleting agent) before cytosolic delivery of aprotinin-AF555. As shown in Figure 3a, only MβCD abolished protein internalization (similar to low-temperature treatment at 4 °C) while endocytosis and pinocytosis inhibitors showed minor reductions, confirming that the protein cell entry largely avoided the classic endocytic pathway.

The non-endocytic pathway was further confirmed by time-lapse imaging (Figure 3b, Supplementary Figure S13, and Movie S1). A gradual increase of diffuse fluorescence in the cytosol and nucleus was observed, as opposed to the initial appearance of punctate spots from endocytosis and subsequent release from endosomes, which is similar to other studies reporting direct cytosolic delivery.^45, 48, 50, 55, 56^ The time it takes for protein entry into the cell cytoplasm is highly heterogenous, likely due to the heterogeneity of cells as well as the cell cycles they are in. Because Coomassie blue alone is not highly fluorescent, dissociation between the noncovalently bound CB-tag and protein was not observed. However, due to the hydrophobicity of the tag, the CB tag is likely left in the cell membrane as the protein slipped inside the cells. Indeed, previously using siRNA and an RNA-tag composed of an RNA binding dye and a cholesterol, siRNA-tag dissociation was observed.^62^

### (d) Cytosolic delivery of primary antibodies.

Next, we proceeded with the delivery of primary antibodies and testing of their functionality in recognizing intracellular targets. We chose three cytoskeleton targets, vimentin intermediate filament, actin microfilament, and microtubule, because of their distinctive filamentous structures from which specific or non-specific binding can be easily discerned. Anti-vimentin monoclonal antibody (V9) was selected to label intermediate filaments. As shown in Figure 4a, when anti-vimentin IgG-AF568 was delivered into HeLa cells, filaments were observed in the cells, indicating that the antibodies were functional and remained correctly folded during delivery. Low-magnification fluorescence imaging also confirmed the delivery efficiency (most of the cells are fluorescent, Figure 4b). To further confirm that the vimentin was localized inside cells, confocal microscopy was used. Z-stack images clearly showed that vimentin was in the cytoplasm (Figure 4d). Interestingly, we also noticed that the intermediate filaments mainly localized near the cell nucleus while fewer filaments were present in the cytosol (Figure 4d, e and Supplementary Movie S2), which is different from the results obtained with fixed cells (Supplementary Figure S14). Similar observations have been made previously with a microinjected monoclonal antibody targeting the intermediate filament which rapidly condensed intermediate filaments to the peri-nuclear region.^63, 64^ These results underline the difference between and significance of imaging live cells over fixed cells. In contrast to the intermediate filaments in fixed cells which cannot reorganize after binding to antibodies, in live cells, intermediate filaments could be crosslinked by IgGs which are bivalent, rapidly reorganizing them to the perinucleus cap.^63, 64^ Moreover, it is noteworthy that, in the cytosol of live cells, specific labeling lasted for several hours offering a window for target tracking if needed. Fluorescent aggregates were observed after overnight incubation (Figure 4c), similar to non-specific secondary antibodies, likely due to antibody degradation and aggregation in the intracellular reducing environment.

To our surprise, unlike the anti-vimentin antibody, monoclonal antibodies against actin and β-tubulin did not show specific labeling although they share a similar structure as the vimentin antibody and they entered the cytosol at high efficiency. These two antibodies showed bright and diffuse fluorescence (Supplementary Figure S15) similar to the secondary IgG shown in Figure 2m. We suspected that this problem could have resulted from antibody binding to the actin/tubulin monomers or oligomers states of, or antibody perturbing the polymeric cytoskeleton structures. A literature survey revealed that depending on the binding epitopes, microtubule and actin microfilament can be disrupted by their antibodies.^65–70^ Compared to intermediate filaments which form more stable bundled structures, actin and tubulin have more dynamic equilibria between the monomeric and polymeric forms. The equilibria can be shifted to the monomeric forms if antibody binding disturbs the interaction between monomers. For example, the binding of large antibodies could allosterically perturb protein structures, and/or sterically interfere with binding. Although this feature is unsuited for imaging cytoskeleton in live cells, potentially it could be desirable for therapeutic applications. For the purpose of imaging actins and microtubules, we employed a new approach, using ligands that stabilize rather than disrupt the polymeric forms.

### (e) Delivery of ligand-protein conjugates for actin and microtubule.

For actin, we selected phalloidin, a cyclic peptide that tightly binds to actin and prevents actin depolymerization. Phalloidin is impermeable to cell membranes, and phalloidin-AF488 conjugate cannot be delivered by the protein CB-tag because it is too small. Therefore, we used the hapten-conjugation strategy commonly used in antibody production to immobilize phalloidin to fluorescently labeled aprotinin using the cysteine-maleimide chemistry (Figure 5a). When phalloidin-aprotinin-AF555 was delivered into cells using the CB tag, actin microfilaments became clearly visible (Figure 5b). The labeling specificity was further proved by adding Cytochalasin D (a toxin that disrupts actin microfilaments) either before adding phalloidin-aprotinin-AF555 to cells or after the cells were already labeled with phalloidin-aprotinin-AF555. Disruption of the actin microfilament was observed regardless of the treatment sequence (Figures 5c & d). In parallel, we also tested whether the biotin-avidin interaction can be utilized because both phalloidin-biotin and fluorescently labeled strept(avidin) are commercially available, and because the phalloidin-protein conjugates can be easily prepared by a simple incubation. When phalloidin-XX-biotin (a commercial phalloidin-biotin conjugate with a relatively short linker) was used to make the conjugate, only diffuse fluorescence inside cells was observed (Figure S16). When phalloidin-PEG12-biotin (a longer spacer between phalloidin and biotin) was used, actin filaments were specifically labeled similar to the covalent conjugate shown in Figure 5f. These results show that i) strept(avidin) maintains its intact structure and binding affinity to biotin in the presence of CB-tag; ii) a long linker is desirable to display the phalloidin moiety without creating significant steric hindrance; and iii) although similar in size, avidin has more accumulation in the nuclei over streptavidin, likely due to its cationic nature.

For microtubules, we explored the same conjugation strategy using Paclitaxel (PTX). PTX binds to the lumen of microtubules and stabilizes them, consequently preventing cell mitosis. While it has been widely prescribed to treat cancer, it can also be used for microtubule imaging.^71, 72^ To combine with our CB-tag technology, PTX was conjugated to Aprotinin-AF555 with PEG linkers of different lengths, PEG_2_ or PEG_12_ (Figure 6a). When they were delivered into HeLa or COS-7 cells, both PTX-aprotinin-AF555 conjugates labeled the microtubule structure, with the longer linker showing slightly better contrast (Figures 6b–c). It is worth mentioning that the PEG linker must be connected to PTX at the 7-O position rather than the 2’-OH position, because 2’-OH is essential for tight binding with tubulin^73^ (In Figure S17, only diffuse fluorescence was observed with PTX functionalized at the 2’-OH position).

Using COS-7 cells which have a thin spreading morphology, we investigated the binding kinetics of PTX-aprotinin-AF555 to microtubules in live cells by time-lapse imaging with 30-s intervals (Figure 6d, and S18). As soon as the fluorescence of PTX-aprotinin-AF555 became detectable inside cells, small fibrils of microtubule started to appear within minutes. Considering the fact that the binding site of PTX is located in the lumen of microtubules, and the relatively large size of PTX-protein, such a fast binding kinetics was somewhat surprising. Previously, using Flutax (a PTX-dye conjugate), it has also been shown that the binding site for PTX is highly accessible.^74^ This accessibility has been attributed to the ability of PTX to rapidly reshape microtubules. PTX modifies the flexibility of microtubules within seconds and changes the number of protofilaments within minutes.^75, 76^

## CONCLUSION

In summary, using the new CB-tag with an optimized protocol we demonstrated that a wide variety of proteins of interest could be transported directly into the cytosol with high efficacy, bypassing endocytosis sequestration. More importantly, delivering fluorescently labeled functional proteins into live cells not only allows direct imaging of intracellular targets of interest similar to IHC on fixed cells, but also enables the observation of cellular responses in real time. This unique feature was exemplified by the time-lapse imaging in live cells during the delivery of PTX-protein conjugates, showing rapid binding kinetics and subsequent disruption of mitochondrial dynamics. Furthermore, we demonstrated that proteins covalently conjugated to peptides and/or small molecules or non-covalently linked through the avidin-biotin interaction could be internalized. Aided by the CB-tag, this kind of membrane permeable conjugates could find numerous applications in cell biology and drug discovery. Some examples include (i) attaching signaling peptides such as a nuclear localization signal to translocate protein cargoes into specific cellular organelles, (ii) preparing bispecific binders for induced proximity, and (iii) degrading targets by attaching E3 ligase ligands for proteolysis targeting chimera (PROTAC). It is worth highlighting that these applications are possible because CB-tag-based protein delivery does not follow the conventional endocytic pathway, instead, protein cargoes are directly transported through the plasma membrane dramatically improving the cargo bioavailability. In addition, the CB-tag approach is also simple, only requiring mixing and incubating. We envision the CB-tag to play important roles in biology research and biologics discovery.

## Supplementary Material

Supplementary Movie S1

Supplementary Movie S2

Supplementary Material

The synthetic diagrams and chemical characterizations of the key compounds, and additional fluorescent cell images can be found online at https://www.sciencedirect.com.

## Figures and Tables

**Figure 1.**
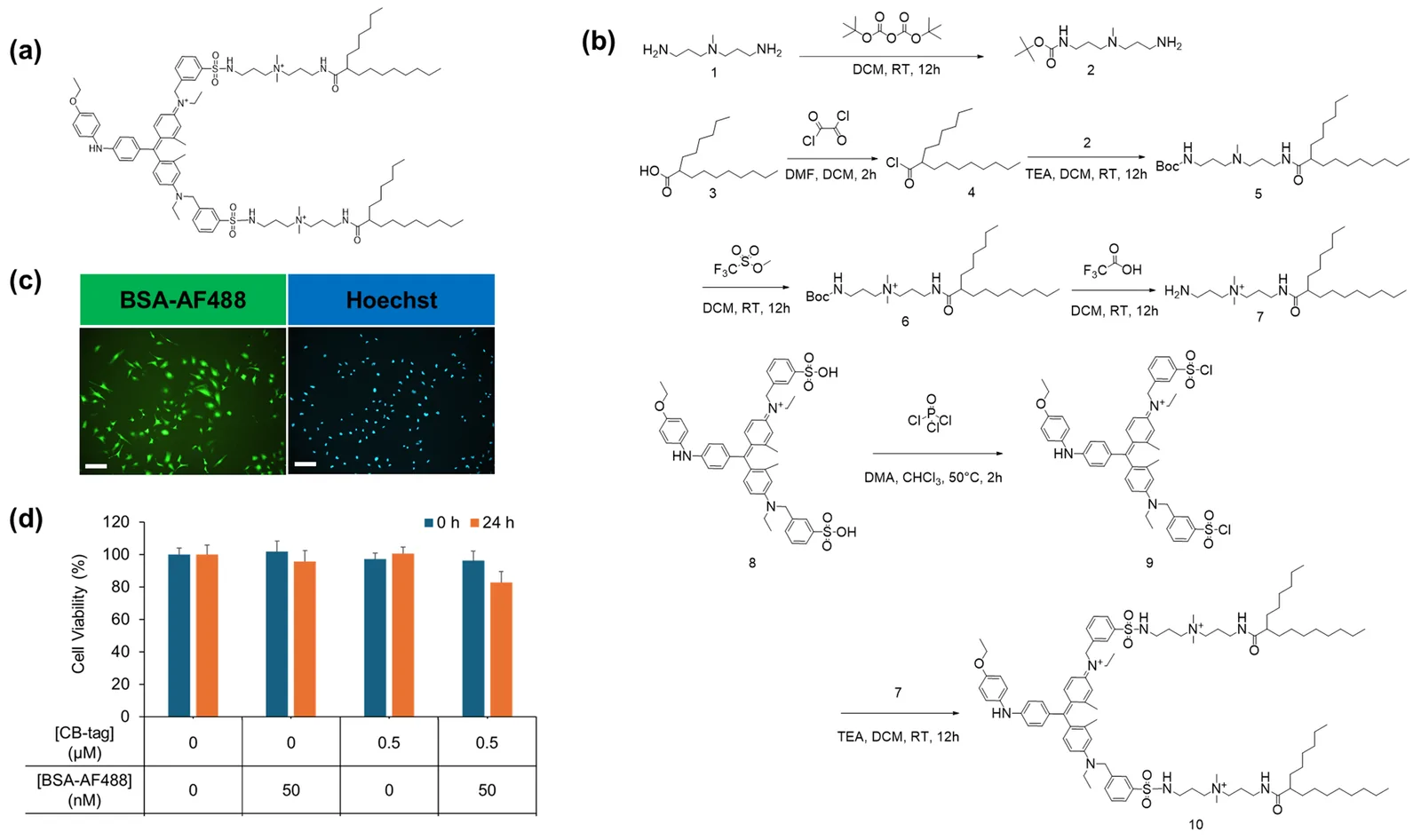
Synthesis and characterization of the new CB-tag. (a) Chemical structure of the CB-Tag. (b) CB-tag synthetic route. Hexyl decanoyl chloride (4) was reacted with the linker 2 to obtain compound 5, whose tertiary amine is subsequently methylated to the quaternary amine (6). After t-Boc deprotection, two copies of 7 were conjugated to CB, resulting in the final product 10. (c) Representative images of HeLa cells after intracellular delivery of BSA-AF488 (BSA concentration 50 nM, Tag ratio 1:10). Scale bar denotes 100 μm. (d) Cell viability evaluation by the Alamar Blue assay of tag-BSA-AF488 complex in HeLa cells immediately after protein delivery (0 h) or after further incubation of 24 h (24 h). ([BSA-AF488] = 50 nM, Protein:Tag=1:10) Results are presented as mean ± standard deviation (S.D.) (n = 4).

**Figure 2.**
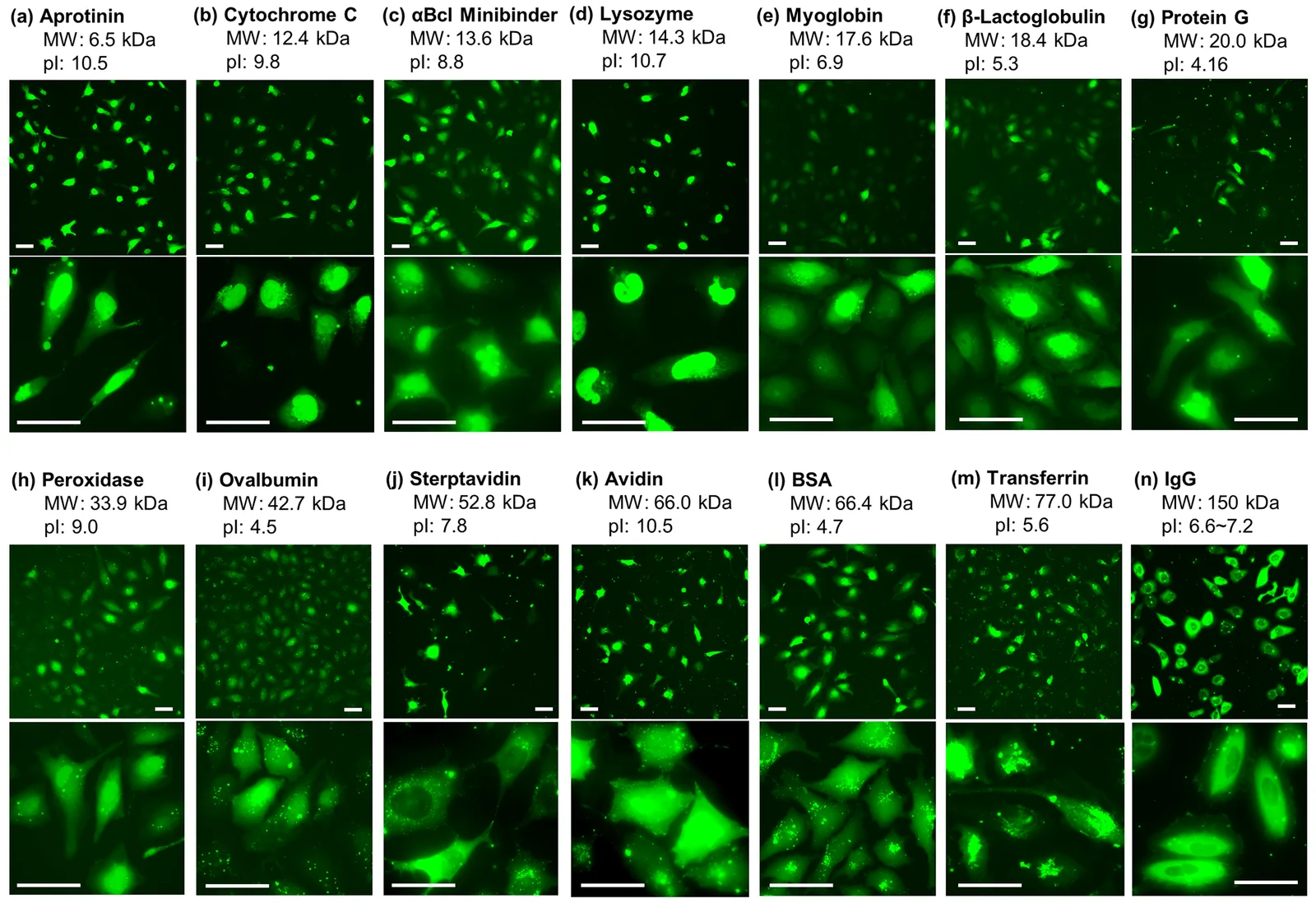
HeLa cells after intracellular delivery of various proteins imaged at low-magnification (10x) to show the cell population with proteins successfully delivered and at high-magnification (40x) to show the protein intracellular distribution. (a) Aprotinin-AF555, 50 nM, Tag ratio=1:2. (b) Cytochrome C-AF555, 100nM, Tag ratio=1:22. (c) αBCL Minibinder-AF488, 100 nM, Tag ratio=1:2. (d) Lysozyme-AF555, 100nM, Tag ratio=1:10. (e) Myoglobin-AF568, 100nM, Tag ratio=1:8. (f) β-Lactoglobulin-AF568, 100 nM, Tag ratio 1:12. (g) Protein G-AF488, 100 nM, Tag ratio 1:6. (h) Horseradish Peroxidase–AF568, 100 nM, Tag ratio 1:1. (i) Ovalbumin-AF568, 100 nM, Tag ratio 1:16. (j) Streptavidin-AF555, 100 nM, Tag ratio 1:6. (k) Avidin-AF555, 100nM, Tag ratio=1:2. (l) BSA–AF488, 50 nM, Tag ratio 1:10. (m) Transferrin-AF555, 100nM, Tag ratio=1:6. (n) Secondary IgG-AF555, 100 nM, Tag ratio 1:10. Scale bar denotes 40 μm.

**Figure 3.**
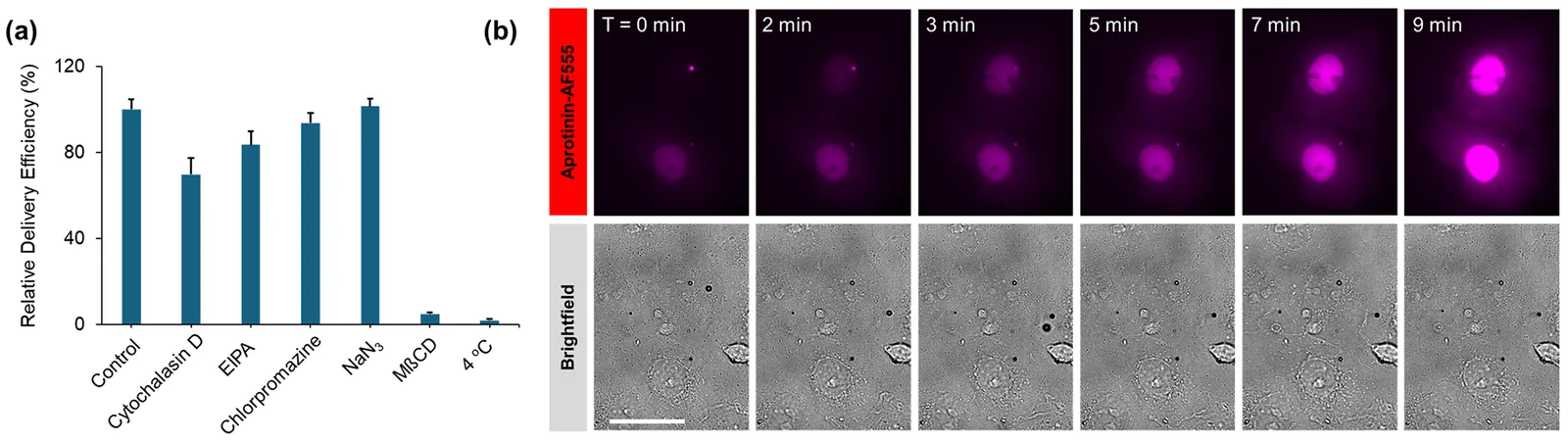
Confirmation of protein cell entry via non-endocytic pathway. (a) Relative percentage of HeLa cells exhibiting diffuse fluorescence in cytosol pretreated with various inhibitors for 1 h, followed by incubating with the complex of Aprotinin-AF555 and CB-tag for 2 h in cell incubator. [Aprotinin-AF555] = 50 nM, Protein:Tag=1:2. (b) Time-lapse imaging of HeLa cells during delivery of Aprotinin-AF555. (100 nM, Tag ratio=1:2, Supplementary Figure S13 and Movie S1) Scale bar denotes 20 μm.

**Figure 4.**
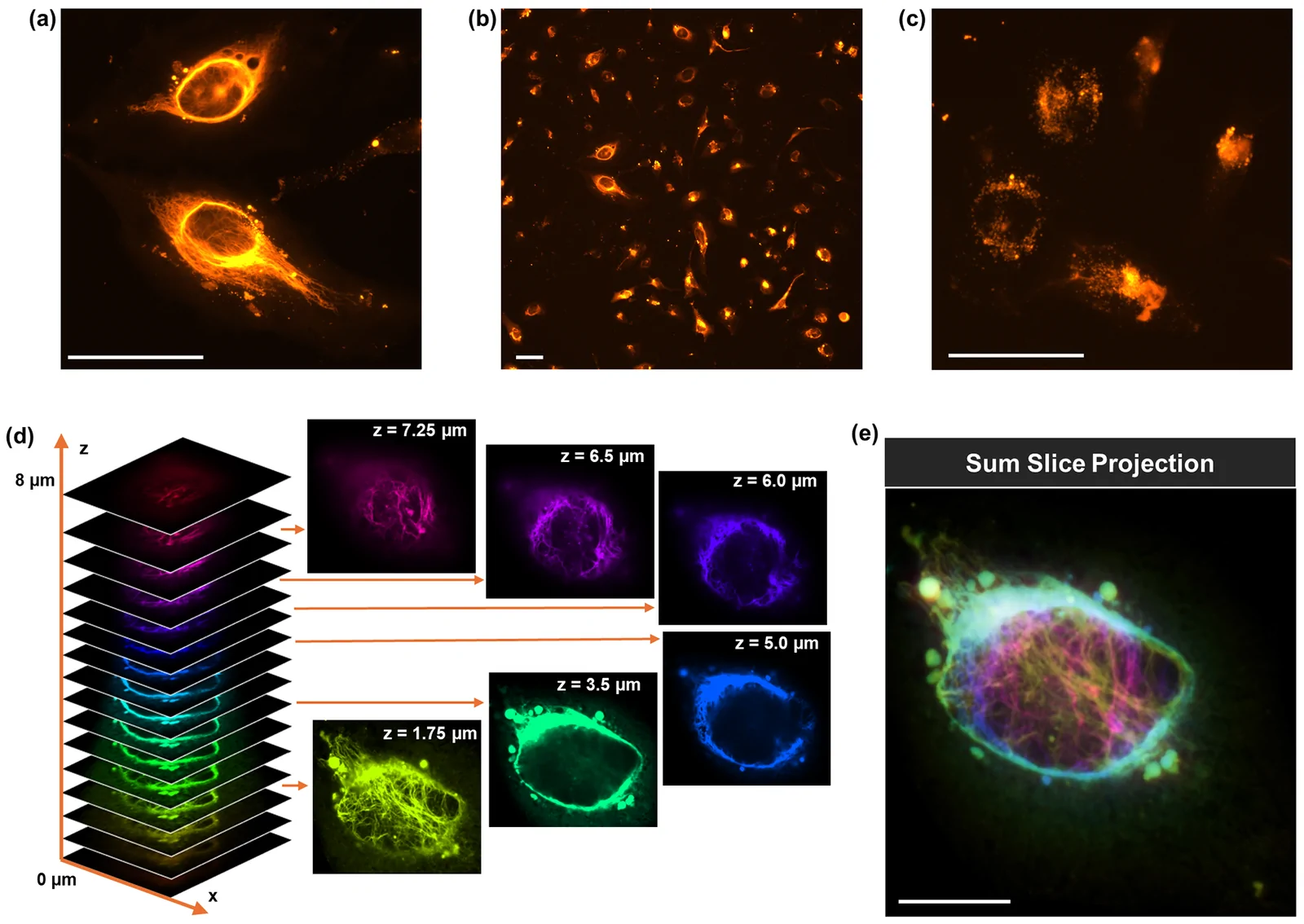
Representative images of HeLa cells after intracellular delivery of anti-Vimentin IgG-AF568. ([IgG-AF568]=50 nM, Tag ratio 1:10) with objective (a) 40X or (b) 10X, or (c) after overnight incubation. Scale bar denotes 40 μm.(d) Representative confocal Z-stack images of HeLa cells after intracellular delivery of anti-Vimentin IgG-AF568. ([IgG-AF568]=50 nM, Tag ratio 1:10) Z-stack slices were depth color-coded, and (e) Sum slices projection was applied to all z-stack slices by ImageJ. (3D projection in Supplementary Movie 2) Scale bar denotes 10 μm.

**Figure 5.**
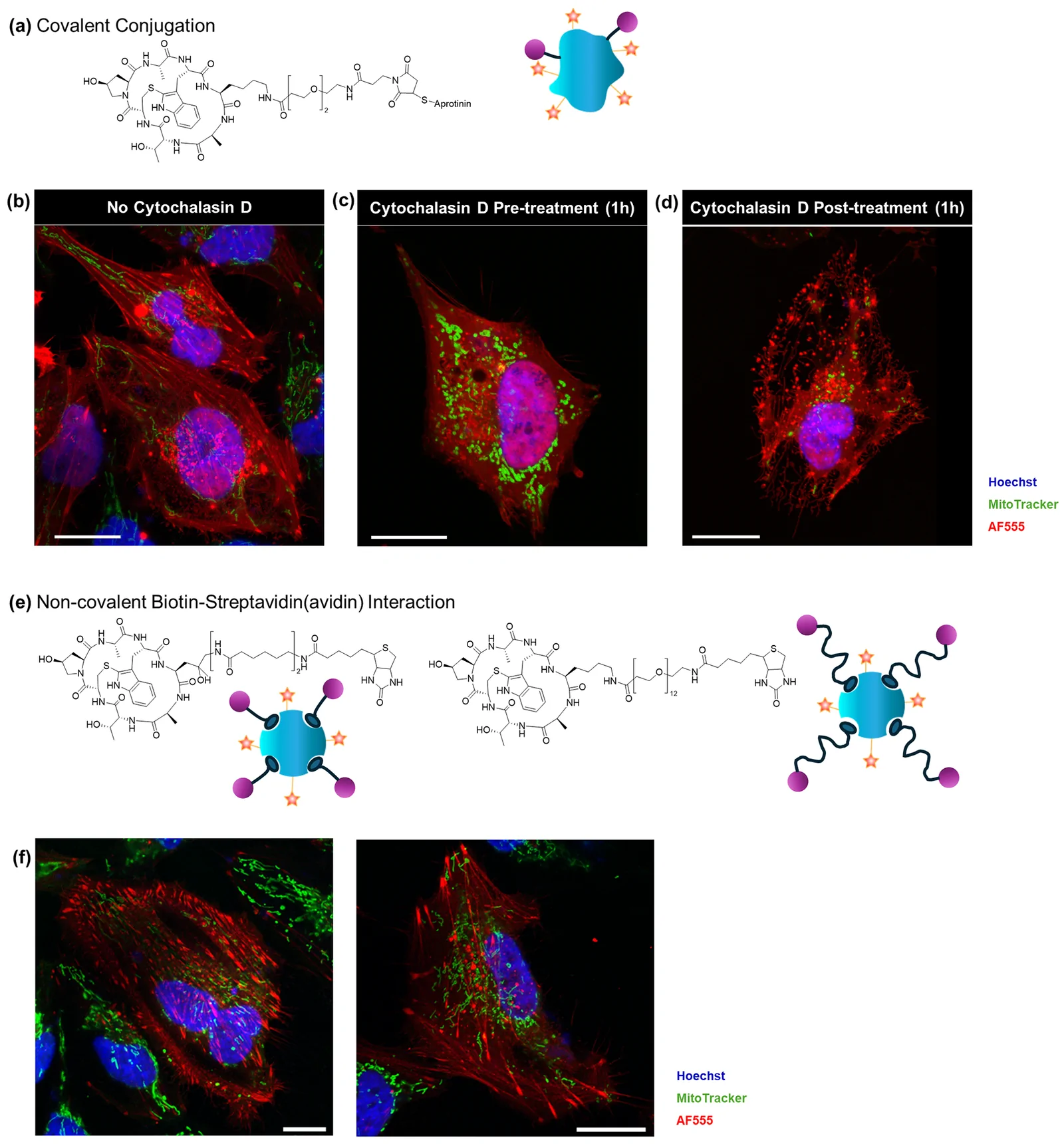
Representative HeLa cells after intracellular delivery of Phalloidin conjugated Aprotinin-AF555. (a) Chemical structure and schematic illustration of Phalloidin-Aprotinin-AF555 covalent conjugates. (b) Representative confocal images of HeLa cells after intracellular delivery of Phalloidin-PEG2-Aprotinin-AF555. (100 nM, Tag ratio=1:2) (c and d) HeLa cells treated with 10 μM Cytochalasin D for 1 h before or after Phalloidin delivery. Scale bar: 20 μm. (e) Chemical structures of Phalloidin-biotin conjugates with various linker length, and schematic illustration of them bound to Avidin-AF555. (f) Representative confocal images of HeLa cells after intracellular delivery of Phalloidin-PEG12-Biotin-Avidin-AF555 (100 nM, Tag ratio=1:2). Scale bar: 20 μm.

**Figure 6.**
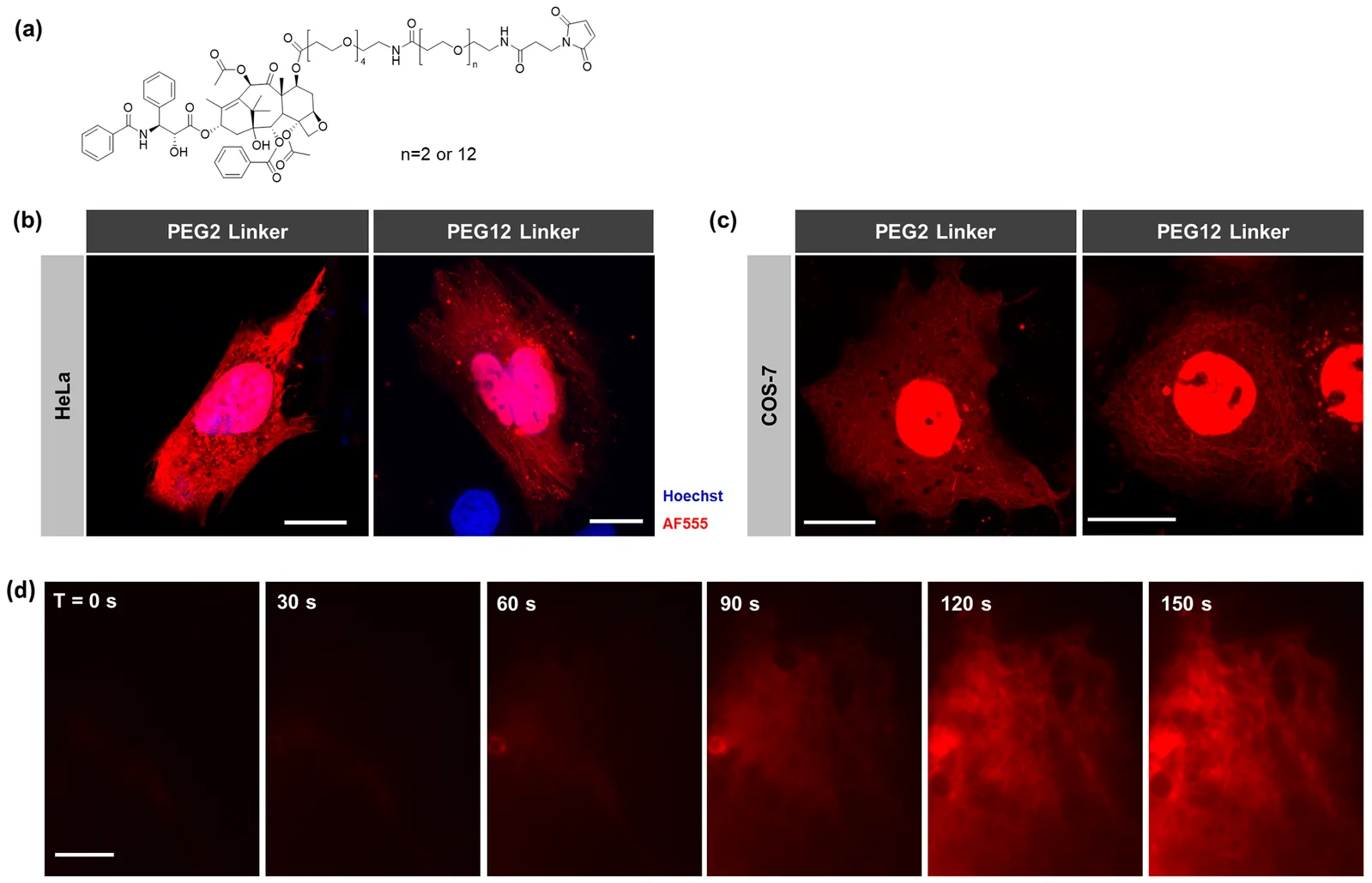
Intracellular delivery of PTX-Aprotinin-AF555 conjugates. (a) Chemical structure of Paclitaxel conjugated at 7-O position with various length of pegylated maleimide linker (n=2 or 12). (b) Representative confocal images of HeLa cells after intracellular delivery of PTX-Aprotinin-AF555 with PEG2, or PEG12 linker. (100 nM, Tag ratio=1:2) Scale bar denotes 20 μm. (c) Representative confocal images of COS-7 cells after intracellular delivery of PTX-Aprotinin-AF555 with PEG2, or PEG12 linker. (100 nM, Tag ratio=1:2) Scale bar denotes 20 μm. (d) Time-lapse imaging of COS-7 cells during delivery of PTX-PEG16-Aprotinin-AF555. (100 nM, Tag ratio=1:2) Zoomed-out view in Figure S18. Fast binding kinetics of PTX-protein conjugate clearly shown by fibril structure in 60 s after cytosolic entrance. Scale bar denotes 5 μm.

## Data Availability

Data will be made available on request.
