# Supplementary material for: Intracellular Delivery of Proteins for Live Cell Imaging
Source: J Control Release. Author manuscript; Available in PMC 2026 Oct 15. (PMC13671267; doi:10.1016/j.jconrel.2025.113651)
Supplement: Supplementary file 1 [file NIHMS2213692-supplement-Supplementary_Material.pdf]

Supporting Information

Table of Contents

1. Synthesis of CB-tag
2. Synthesis of Phalloidin Conjugates
3. Synthesis of Paclitaxel Conjugates
4. Supplementary Figure S1. ^{1}H-NMR spectrum of compound **5**.
5. Supplementary Figure S2. ^{13}C-NMR spectrum of compound **5**.
6. Supplementary Figure S3. ESI-MS spectrum of compound **5**.
7. Supplementary Figure S4. ESI-MS spectrum of compound **6.**
8. Supplementary Figure S5. ESI-MS spectrum of compound **7.**
9. Supplementary Figure S6. ESI-MS spectrum of compound **9**.
10. Supplementary Figure S7. ESI-MS spectrum of compound **10** (CB-Tag).
11. Supplementary Figure S8. ^{1}H NMR spectrum of compound **10** (CB-Tag).
12. Supplementary Figure S9. ESI-MS spectrum of Phalloidin-PEG2-Maleimide.
13. Supplementary Figure S10. ESI-MS spectrum of Phalloidin-PEG12-Biotin.
14. Supplementary Figure S11. ESI-MS spectrum of PTX-PEG6-Maleimide.
15. Supplementary Figure S12. ESI-MS spectrum of PTX-PEG16-Maleimide.
16. Supplementary Figure S13. Fluorescence intensity change of the cell in Figure 3B as a function of time.
17. Supplementary Figure S14. Representative image of fixed HeLa cells stained with anti-vimentin IgG-AF568.
18. Supplementary Figure S15. Representative fluorescence images of HeLa cells after intracellular delivery of anti-ß-Tubulin IgG-AF555 or anti-Actin IgG-AF555.
19. Supplementary Figure S16. Representative fluorescence images of HeLa cells after intracellular co-delivery of Phalloidin-XX-Biotin with Avidin-AF555 or Streptavidin-AF555.
20. Supplementary Figure S17. Intracellular delivery of PTX-Aprotinin-AF555.
21. Supplementary Figure S18. Zoomed-out view of **Figure 6d**.

A. Synthesis of CB-tag

(a) Tert-butyl N-{3-[(3-aminopropyl)(methyl)amino]propyl}carbamate (2)

Bis(3-aminopropyl)(methyl)amine (**1**) (72.6 g, 500 mmol) was dissolved in 500 ml of dry dichloromethane (DCM). The reaction was cooled to 0 °C, and di-tert-butyl decarbonate (11.5 ml, 50 mmol) was slowly added dropwise over 24 hours. After the addition was complete, the reaction mixture was allowed to warm up to RT and stirred for 12 hours. To remove access compound **1**, the reaction mixture was washed five times with water. The organic phase was dried with anhydrous Na_2SO_4 and concentrated in vacuo. The product (**2**) was used in the next step without further purification.

(b) 2-hexyldecanoyl chloride (4)

2-hexyldecanoic acid (**3**) (3.0 g, 11.7 mmol) was dissolved in dry DCM (50 ml), and cooled in an ice bath. Oxalyl chloride (1.09 ml, 12.9 mmol) was slowly added dropwise. 5 drops of DMF were added as a catalyst. The reaction proceeded until the bubbling ceased. The reaction mixture was concentrated in vacuo, producing a yellowish oil. The crude product (**4**) was used in the next step without further purification.

(c) tert-butyl N-(3-{[3-(2-hexyldecanamido)propyl](methyl)amino}propyl)carbamate (5)

Compound **2** (3.17 g, 12.9 mmol) was dissolved in dry DCM (30 ml), stirred, and cooled in an ice bath. To this solution, 20 ml of dry DCM solution of 2-hexyldecanoyl chloride (4) (3.22 g, 11.7 mmol) was added, followed by triethylamine (1.8 ml, 12.9 mmol). The reaction mixture was allowed to warm up to RT and stirred overnight. The reaction mixture was washed with water three times, and the organic phase was separated, dried with anhydrous Na_2SO_4, and concentrated in vacuo. The crude product was loaded onto silica gels and purified by flash column chromatography (DCM, and then, 10% MeOH in DCM) yielding compound **5** (5.61g, 99.11% yield).

^{1}H NMR (500 MHz, $CDCl_3$): δ ppm 6.57 (1H, bs), 5.14, (1H, bs), 3.30 (2H, m), 3.15 (2H, m), 2.36 (4H, m), 2.16 (3H, s), 1.96 (1H, m), 1.68-1.48 (6H, m), 1.41 (9H, s), 1.34 (2H, m), 1.22 (20H, s), 0.84 (6H, m)

^{13}C NMR (500 MHz, CDCl3): δ ppm 176.07, 156.05, 78.97, 56.07, 55.72, 48.11, 41.80, 39.34, 38.10, 33.17, 31.86, 31.78, 29.75, 29.54, 29.39, 29.28, 28.42, 27.77, 27.72, 27.38, 26.46, 22.65, 22.61, 14.07.

ESI-MS m/z: $[M + H]^+$ calcd. for $C_{28}H_{57}N_3O_3$, 484.4; found, 484.4.

(d) tert-butyl N-(3-{[3-(2-hexyldecanamido)propyl]dimethylazaniumyl}propyl)carbamate (6)

Compound **5** (5.61 g, 11.596 mmol) was dissolved in anhydrous DCM (100 ml) under N_2 atmosphere. The solution was stirred and cooled to 0 °C in an ice bath. MeOTf (2.54 mL, 23.19 mmol) was slowly added dropwise to the solution. After the addition was complete, the reaction mixture was allowed to warm up to RT and stirred for 12 hours. The reaction was cooled to 0 °C in an ice bath, and then 30ml of deionized water was slowly added to quench excess MeOTf. After stirring for 1 hour, the solvent was evaporated under reduced pressure. The crude product **6** was used in the next step without further purification.

ESI-MS m/z: $[M]^+$ calcd. for $C_{29}H_{60}N_3O_3$, 498.5; found, 498.3.

(e) (3-aminopropyl)[3-(2-hexyldecanamido)propyl]dimethylazanium (7)

Compound **6** (5.74 g, 11.5 mmol) was dissolved in dry DCM (100 ml). The solution was stirred and cooled to 0 °C in an ice bath. TFA (5 ml, 65.8 mmol) was slowly added to the solution. The reaction mixture was allowed to warm up to RT and stirred overnight. The reaction mixture was concentrated in vacuo. The crude product **7** was used in the next step without further purification.

ESI-MS m/z: $[M]^+$ calcd. for $C_{24}H_{52}N_3O$, 398.41; found, 398.4.

(f) CB-tag (10)

Coomassie blue G-250 (**8**) (100 mg, 0.117 mmol) was dissolved in anhydrous DMA (10 ml) and chloroform (15 ml) under N_2 atmosphere. To the solution, phosphorus(V) oxychloride (100 µL, 1.05 mmol) was added dropwise. The reaction mixture was refluxed for 2 hours at 50 °C and then cooled to RT. Chilled dry ethyl ether (100 ml) was added to the reaction mixture to precipitate the product. The precipitated compound **9** was filtered, washed with ether, resuspended in 10 ml of anhydrous DCM, and washed with water two times to quench excessive residual phosphrus(V) oxychloride while the pH of water phase was adjusted to neutral by adding sodium bicarbonate solution. Conversion to sulfonyl chloride was confirmed by MS. The organic phase was separated, dried with anhydrous Na_2SO_4, and added to the solution of compound **7** (0.585 mmol) in 10 ml anhydrous DCM, followed by triethylamine (100 µl). The reaction mixture was stirred at RT overnight. The reaction mixture was washed with water three times, and the organic phase was separated, dried with anhydrous Na_2SO_4, and concentrated in vacuo. The crude product was loaded onto silica gels and purified by flash column chromatography (90:10:1 DCM:MeOH:NH_4OH, and then, increase MeOH and NH_4OH) yielding the compound **10** (83mg, assuming two CF_3SO^- counter ions, 37.6% yield).

^1H NMR (500 MHz, $CDCl_3$): δ (ppm) = (CD_3OD, 300 MHz) δ ppm 7.91-7.71 (4H, m, Ar-H), 7.65-7.42 (5H, m, Ar-H), 7.33-7.17 (4H, m, Ar-H), 7.10-6.94 (5H, m, Ar-H), 6.87-6.70 (4H, m, Ar-H), 4.90 (4H, s, Ar-CH_2), 4.14-3.98 (2H, q, $CH_3C\underline{H}_2$-O), 3.81-3.68 (4H, q, $CH_3C\underline{H}_2$-N), 3.50-3.01 (28H, m, $(C\underline{H}_3)_2$-N^+-$(C\underline{H}_2CH_2C\underline{H}_2)_2$), 2.24-1.80 (10H, m, $(CH_3)_2$-N^+-$(CH_2C\underline{H}_2)_2$, $C\underline{H}$-$(CH_2)_2$ of

hexyldecanoyl), 2.03 (6H, s, CH_3-Ar), 1.65-1.46 (8H, m, CH-(C$\underline{H}_2$)$_2$ of hexyldecanoyl), 1.46-1.34 (9H, m, C$\underline{H}_3$$CH_2$-O, C$\underline{H}_3$$CH_2$-N), 1.34-1.18 (40H, m, CH_2 of hexyldecanoyl), 0.98-0.80 (12H, m, CH_3 of hexyldecanoyl)
ESI-MS m/z: $[M - 2H^+]^+$ calcd. for $C_{95}H_{148}N_9O_7S_2$, 1592.11; found, 1592.1, $[M + CF_3SO^- - H^+]^+$ calcd. for $C_{96}H_{149}F_3N_9O_{10}S_3$, 1742.06; found, 1742.0. $[M + 2\times CF_3SO^-]^+$ calcd. for $C_{97}H_{150}F_6N_9O_{13}S_4$, 1892.02; found, 1891.9.

B. Synthesis of Phalloidin Conjugates

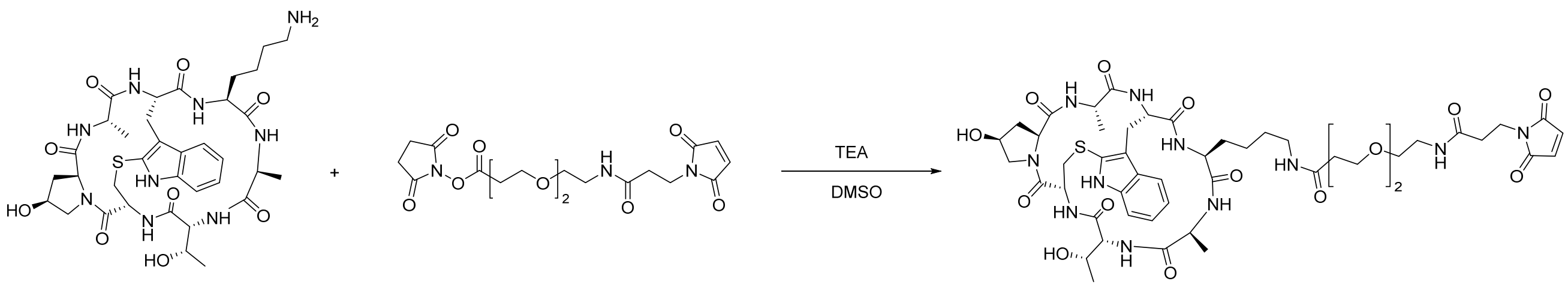

(a) Phalloidin-PEG2-Maleimide

Phalloidin-lysine (0.1 mg, 113 nmol) was dissolved in anhydrous DMSO (113 µl). Afterward, SM(PEG)2 (52.9 µg, 124 nmol) and TEA (0.016 µl, 113 nmol) were added, and the reaction mixture was stirred overnight at RT.
ESI-MS m/z: $[M + H^+]^+$ calcd. for : $C_{49}H_{68}N_{11}O_{15}S$, 1082.45; found, 1082.4, $[M + Na^+]^+$ calcd. for : $C_{49}H_{67}N_{11}O_{15}S$ Na, 1104.44; found, 1104.5.

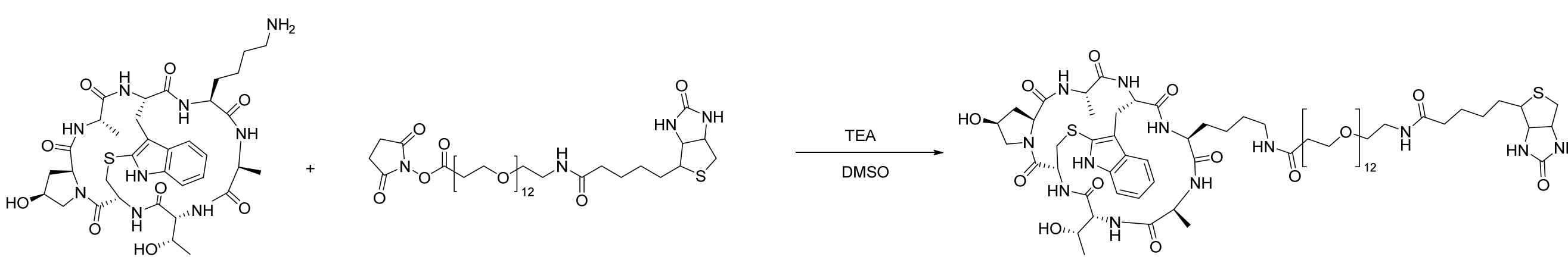

(b) Phalloidin-PEG12-Biotin

Phalloidin-lysine (0.1 mg, 113 nmol) was dissolved in anhydrous DMSO (113 µl). Afterward, NHS-PEG12-Biotin(0.11 mg, 113 nmol) and TEA (0.016 µl, 113 nmol) were added, and the reaction mixture was stirred overnight at RT.
ESI-MS m/z: $[M + H^+]^+$ calcd. for : $C_{72}H_{117}N_{12}O_{24}S_2$, 1597.77; found, 1597.6, $[M + Na^+]^+$ calcd. for : $C_{72}H_{116}N_{12}O_{24}Na$, 1619.76; found, 1619.6.

C. Synthesis of Paclitaxel Conjugates

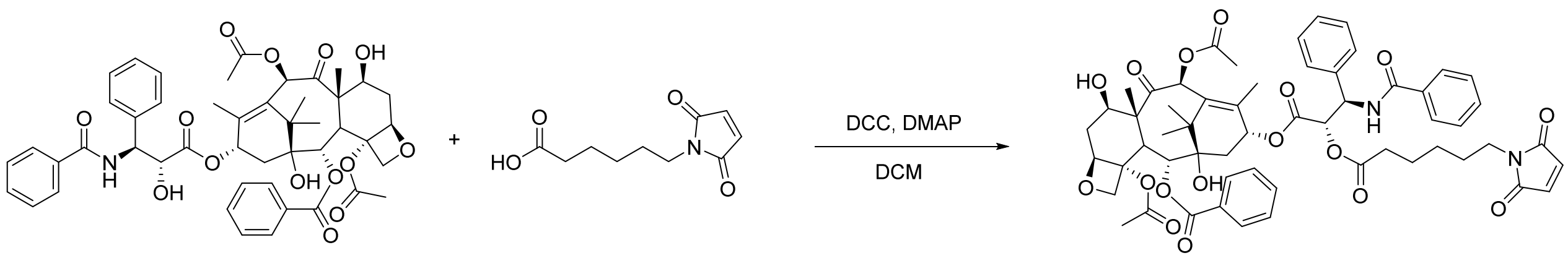

(c) Paclitaxel-Hexyl-Maleimide

Paclitaxel-hexyl-maleimide was synthesized according to the previously reported protocol (*Drug Deliv.* 25, 807-814, 2018).

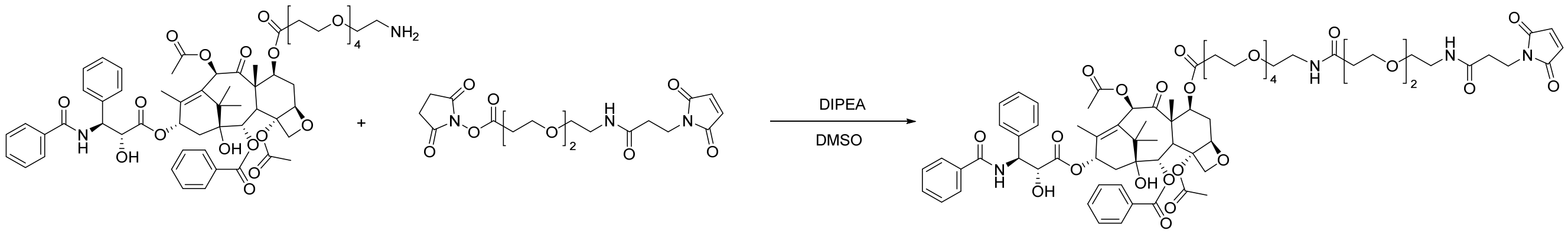

(d) Paclitaxel-PEG6-Maleimide

7-O-(Amino-PEG4)- paclitaxel (0.11 mg, 100 nmol) was dissolved in anhydrous DMSO (0.1 ml). Afterward, SM(PEG)2 (63.8 μg, 150 nmol) and DIPEA (0.017 μl, 100 nmol) were added, and the reaction mixture was stirred overnight at RT.

ESI-MS m/z: $[M + H^+]^+$ calcd. for : $C_{72}H_{91}N_4O_{25}$, 1411.59; found, 1411.4, $[M + Na^+]^+$ calcd. for : $C_{72}H_{90}N_4O_{25}Na$, 1433.58; found, 1433.5.

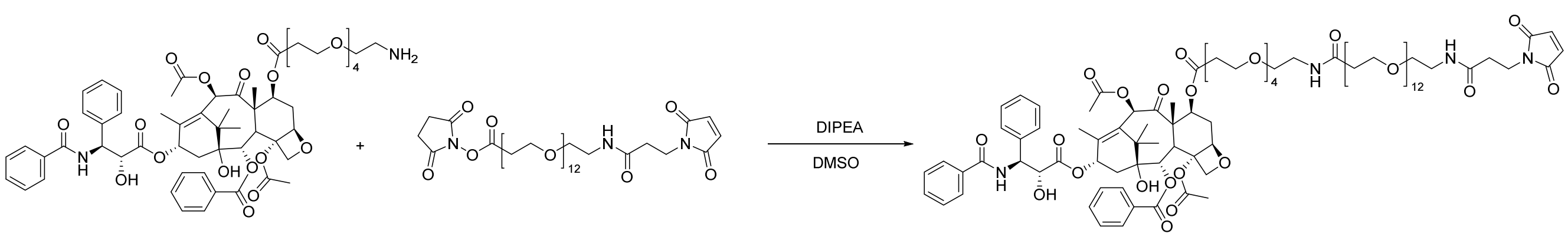

(e) Paclitaxel-PEG16-Maleimide

7-O-(Amino-PEG4)- paclitaxel (0.11 mg, 100 nmol) was dissolved in anhydrous DMSO (0.1 ml). Afterward, SM(PEG)12 (0.13 mg, 150 nmol) and DIPEA (0.017 μl, 100 nmol) were added, and the reaction mixture was stirred overnight at RT.

ESI-MS m/z: $[M + H^+]^+$ calcd. for : $C_{92}H_{131}N_4O_{35}$, 1852.85; found, 1852.5, $[M + Na^+]^+$ calcd. for : $C_{92}H_{130}N_4O_{35}Na$, 1873.84; found, 1873.6.

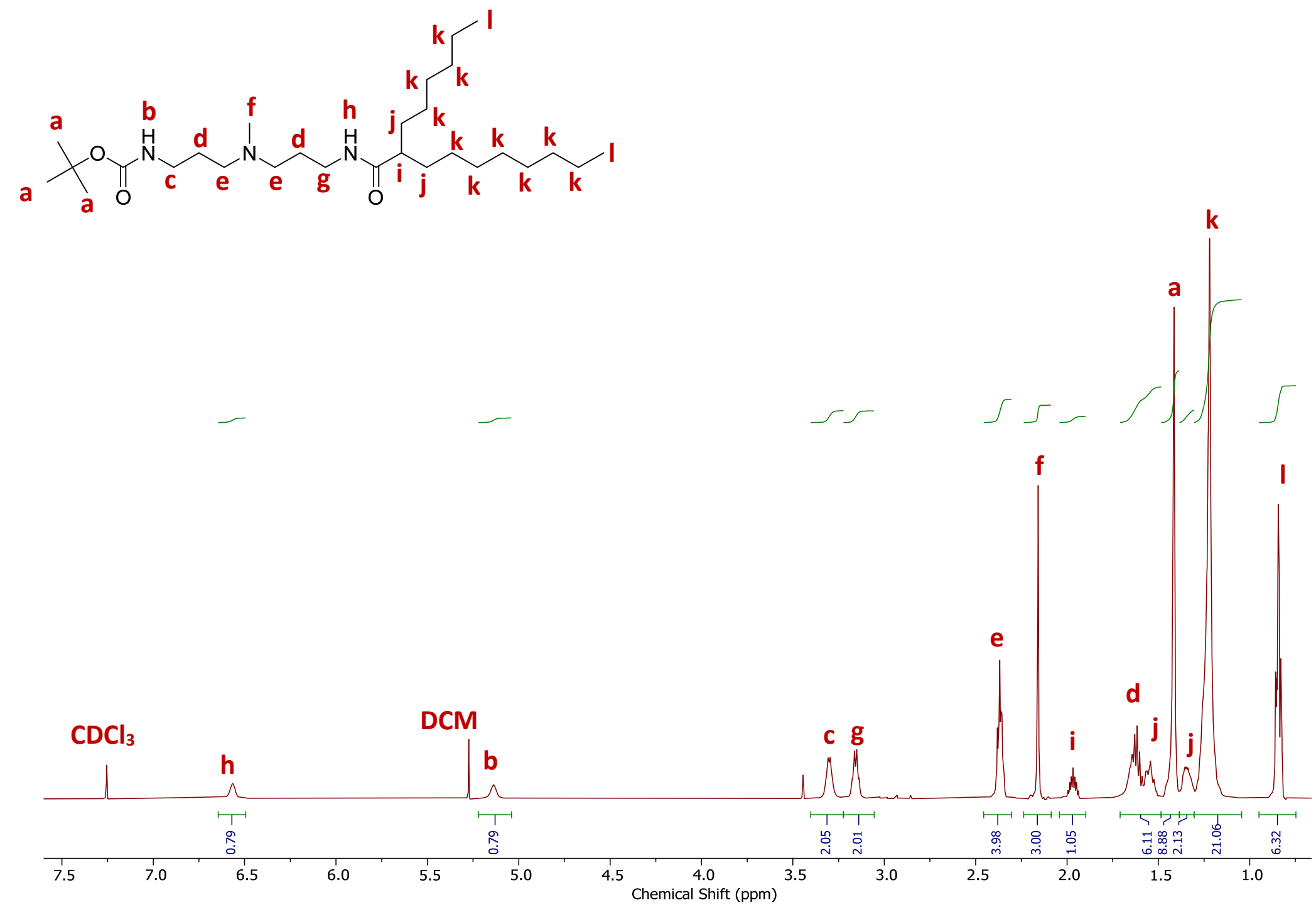

Figure S1. ^{1}H-NMR spectrum of compound **5**. NMR was measured in $CDCl_3$.

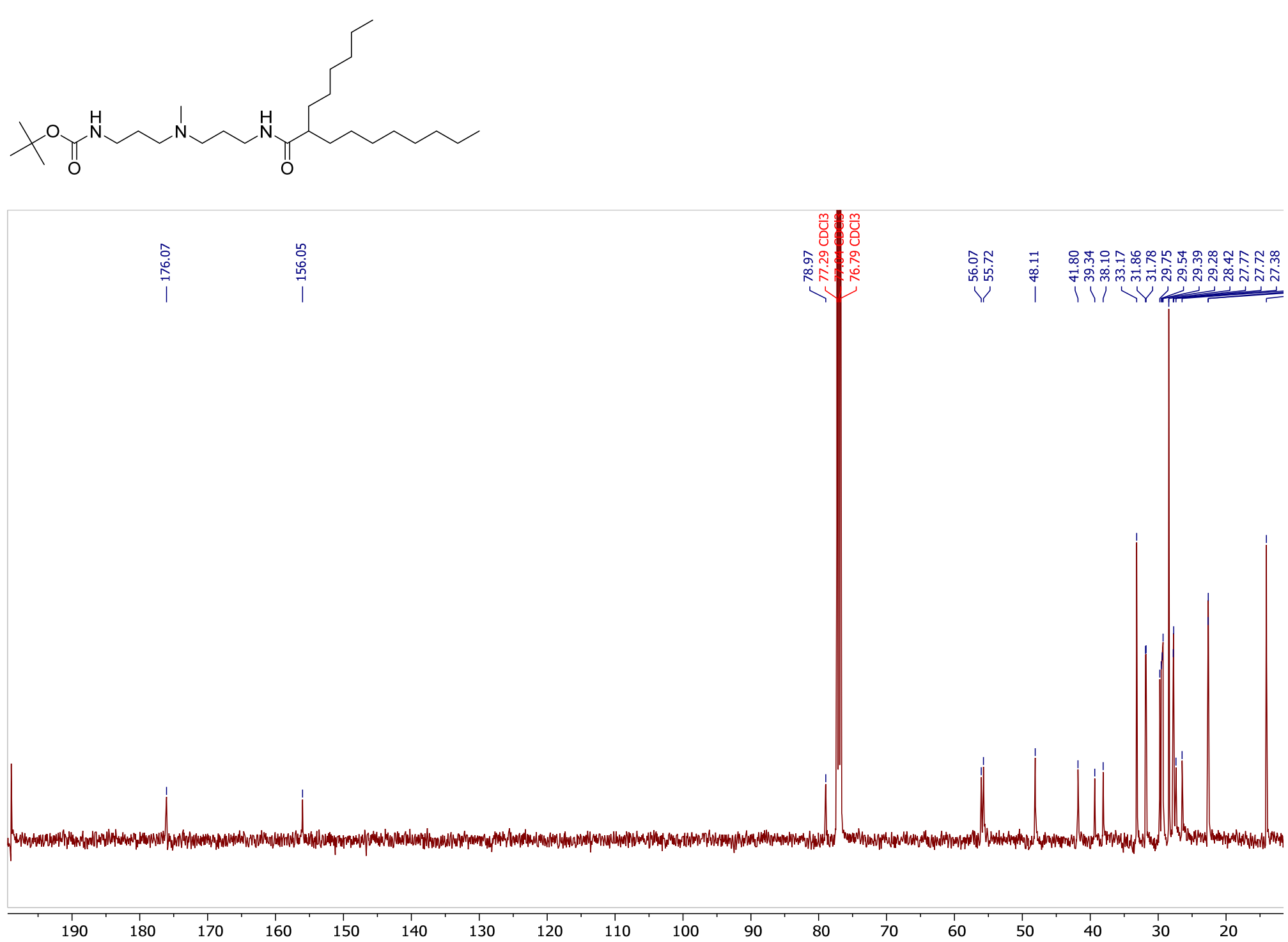

Figure S2. ^{13}C-NMR spectrum of compound **5**. NMR was measured in $CDCl_3$.

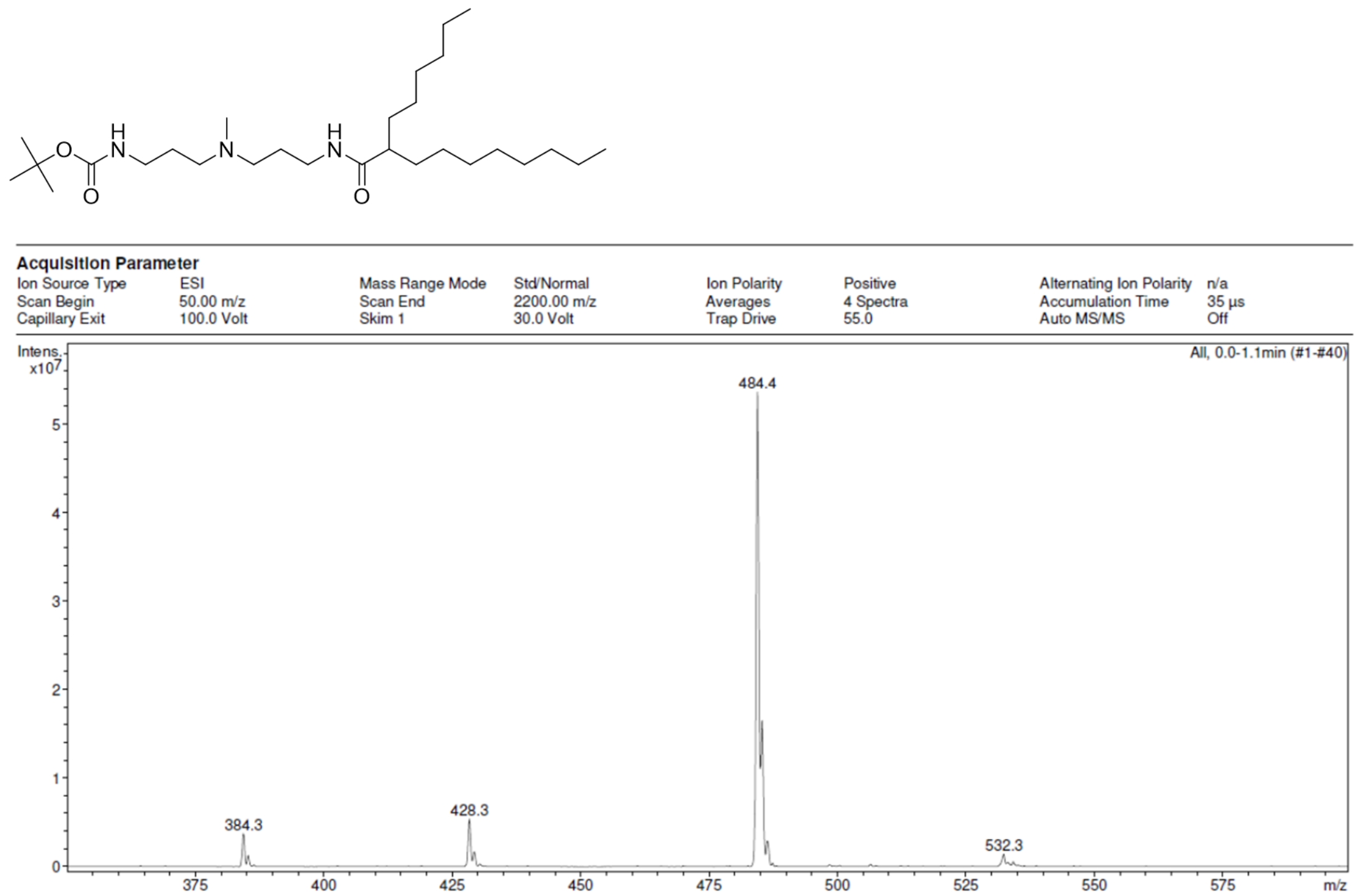

Figure S3. ESI-MS spectrum of compound **5**. ESI-MS m/z: $[M + H]^+$ calcd. for $C_{28}H_{57}N_3O_3$, 484.4; found, 484.4.

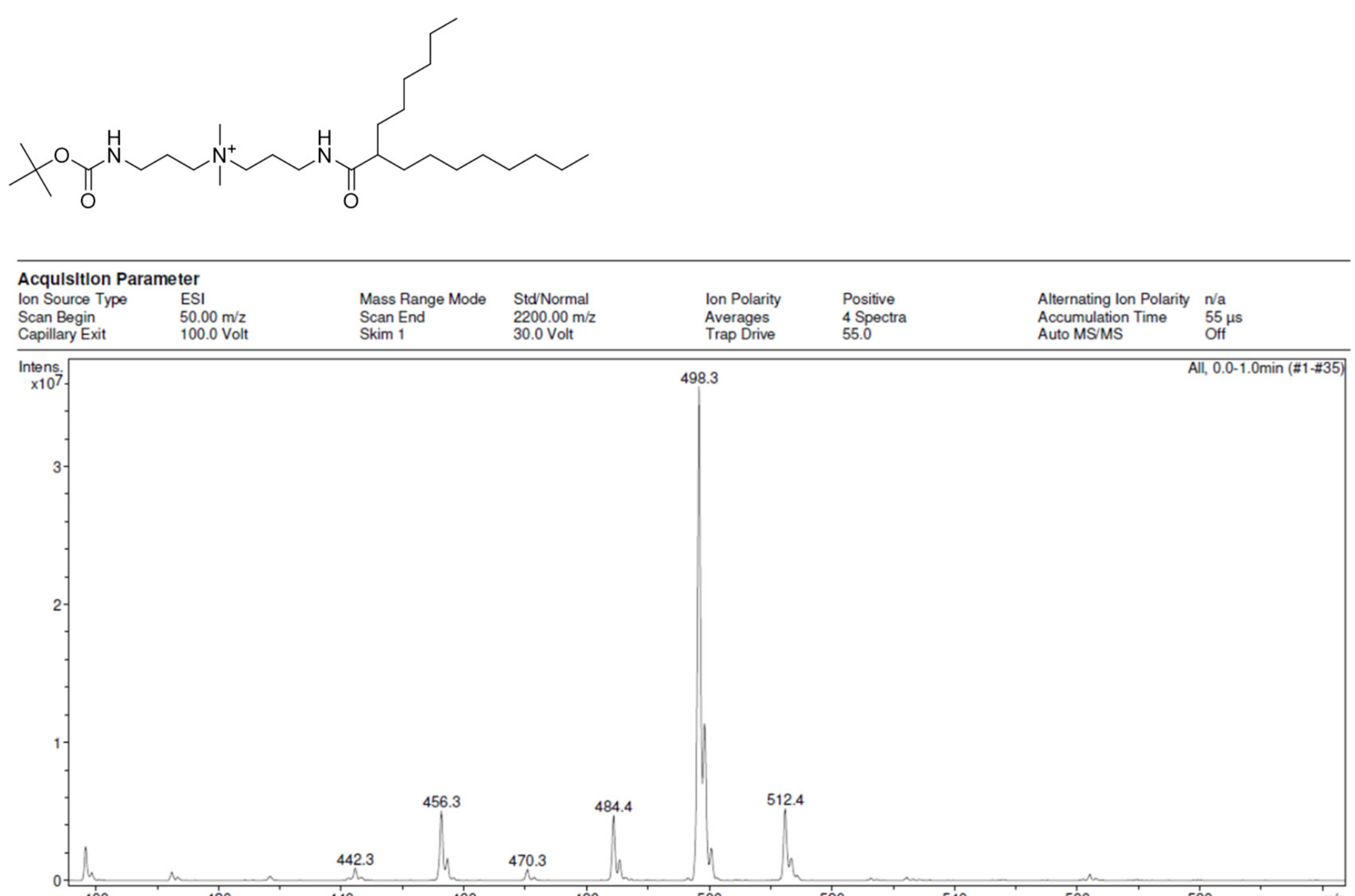

Figure S4. ESI-MS spectrum of compound **6**. ESI-MS m/z: $[M]^+$ calcd. for $C_{29}H_{60}N_3O_3^+$, 498.5; found, 498.3.

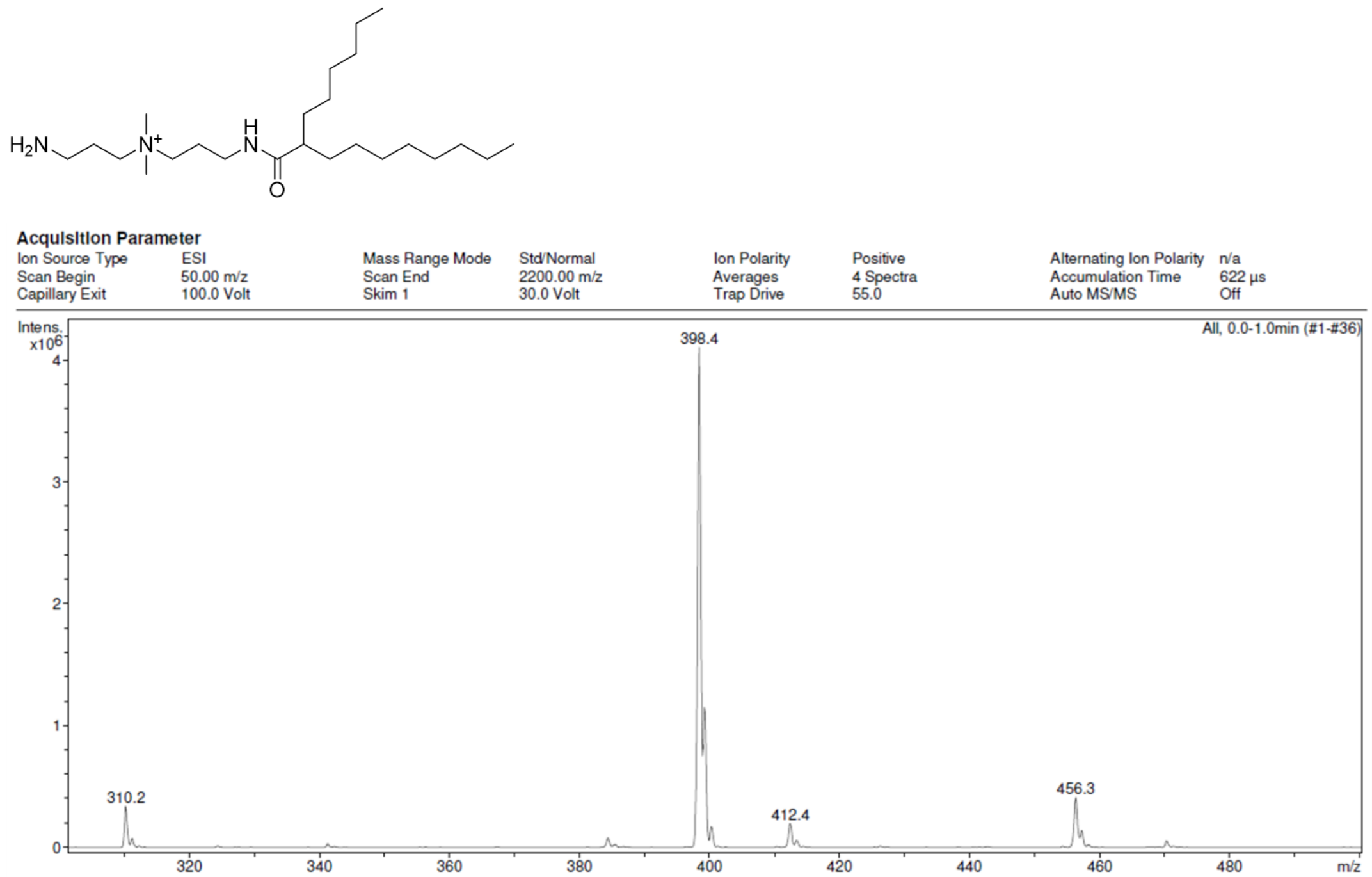

Figure S5. ESI-MS spectrum of compound **7**. ESI-MS m/z: $[M]^+$ calcd. for $C_{24}H_{52}N_3O^+$, 398.41; found, 398.4.

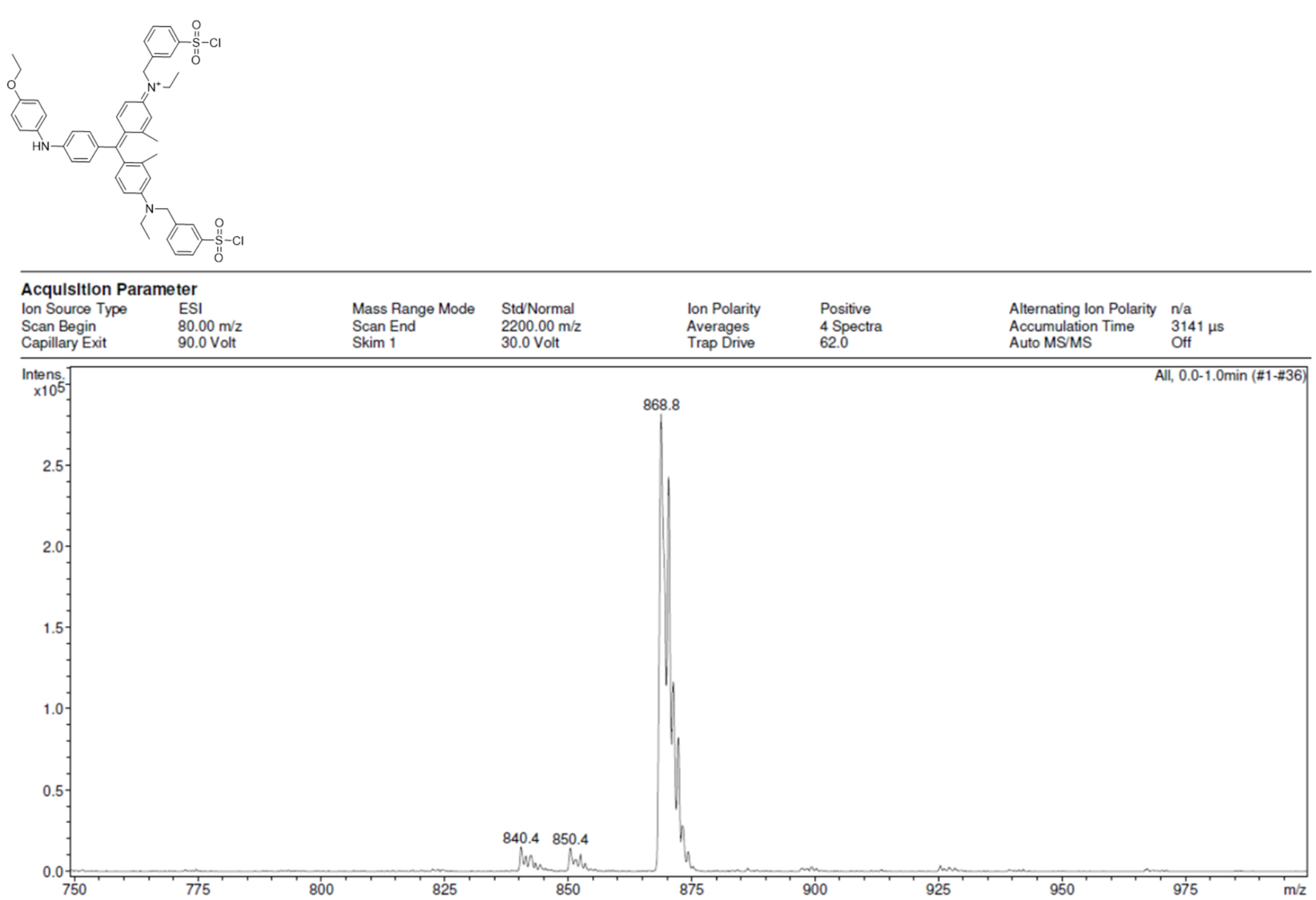

Figure S6. ESI-MS spectrum of compound **9**. ESI-MS m/z: $[M]^+$ calcd. for $C_{47}H_{48}Cl_2N_3O_5S_2^+$,868.24; found, 868.8.

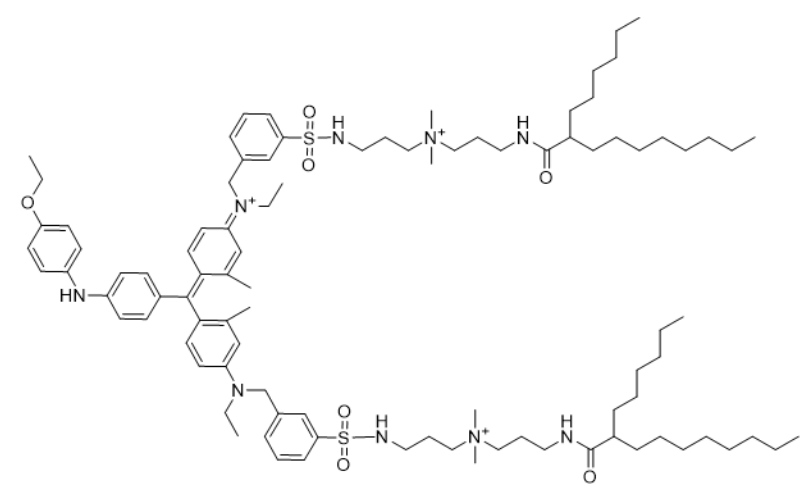

(a)

Acquisition Parameter

Ion Source Type	ESI	Mass Range Mode	Std/Normal	Ion Polarity	Positive	Alternating Ion Polarity	n/a
Scan Begin	50.00 m/z	Scan End	2200.00 m/z	Averages	4 Spectra	Accumulation Time	671 µs
Capillary Exit	160.3 Volt	Skim 1	67.4 Volt	Trap Drive	118.5	Auto MS/MS	Off

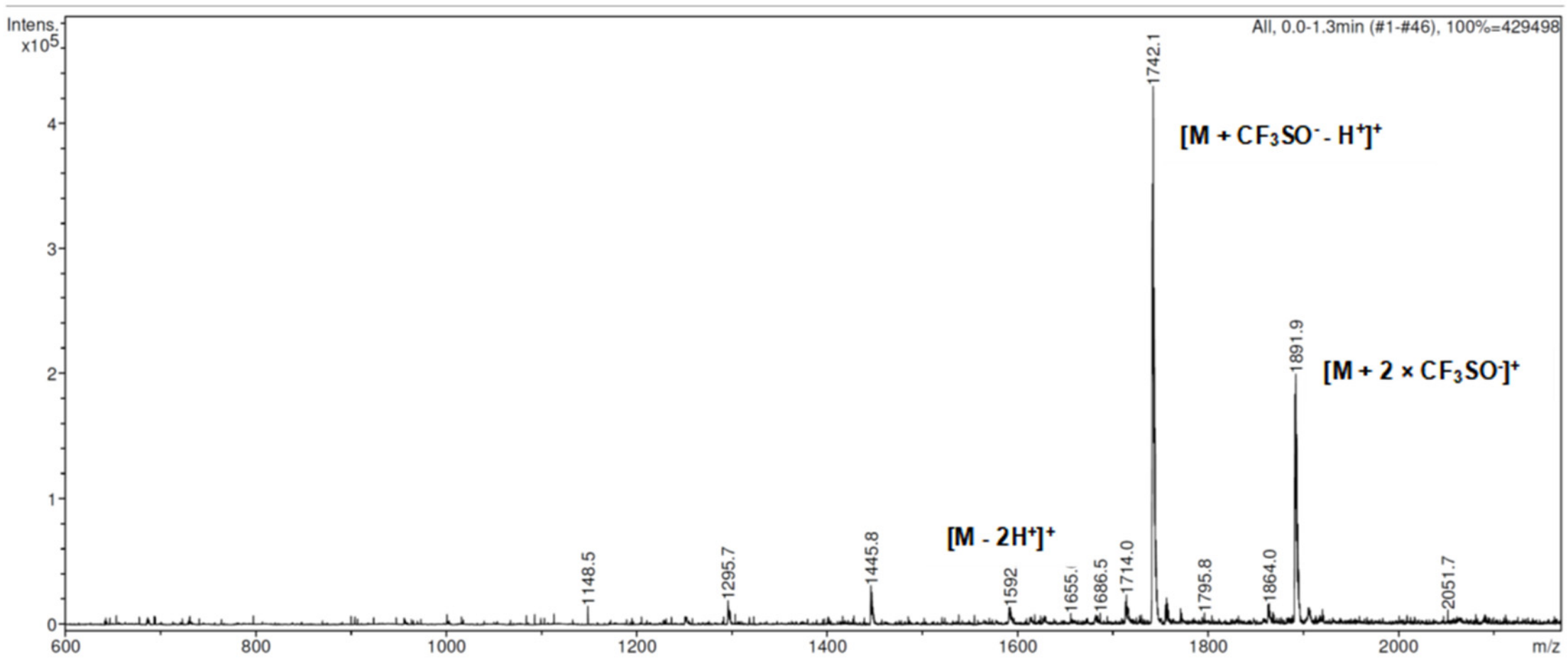

[M + CF$_3$SO$^-$ - H$^+$]$^+$

(b)Observed in MS

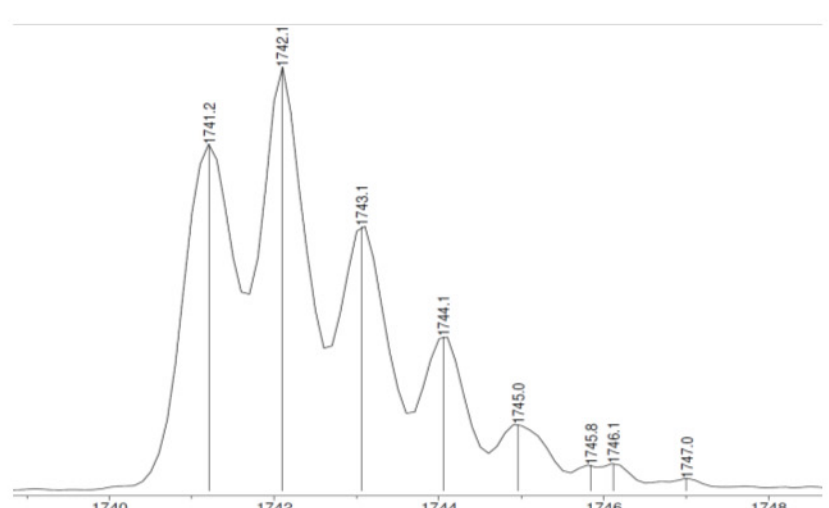

(c) Predicted

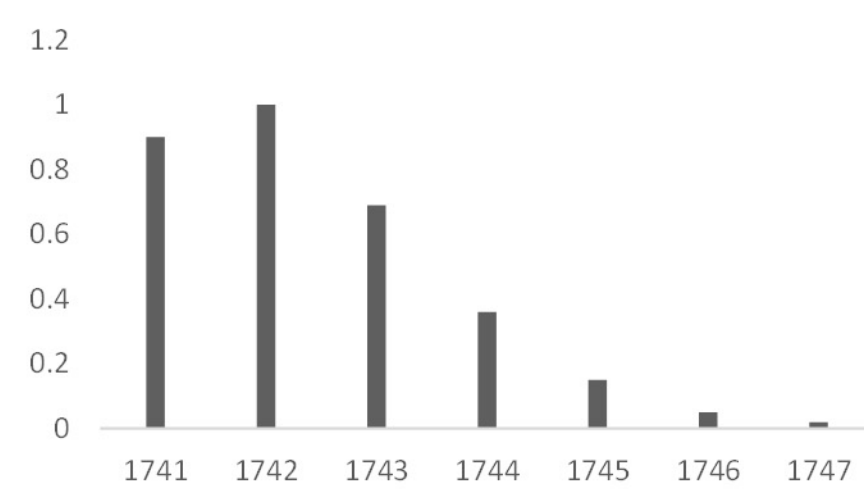

[M + 2 × CF$_3$SO$^-$]$^+$

(d)Observed in MS

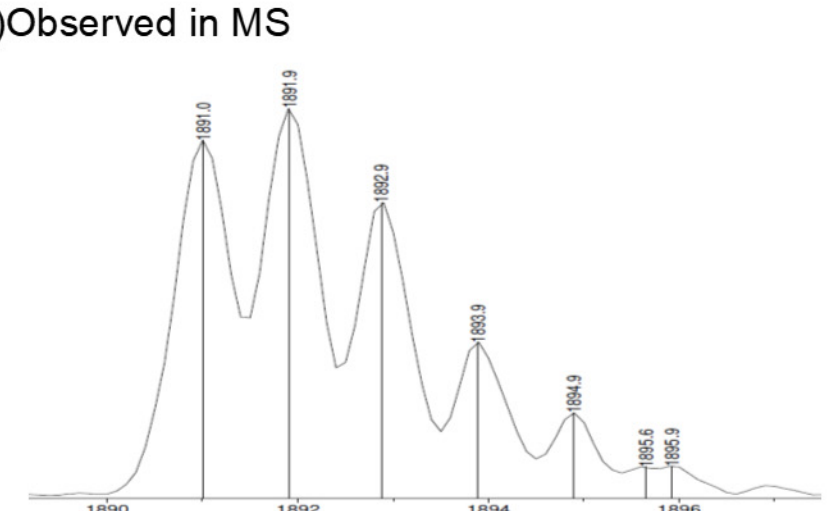

(e) Predicted

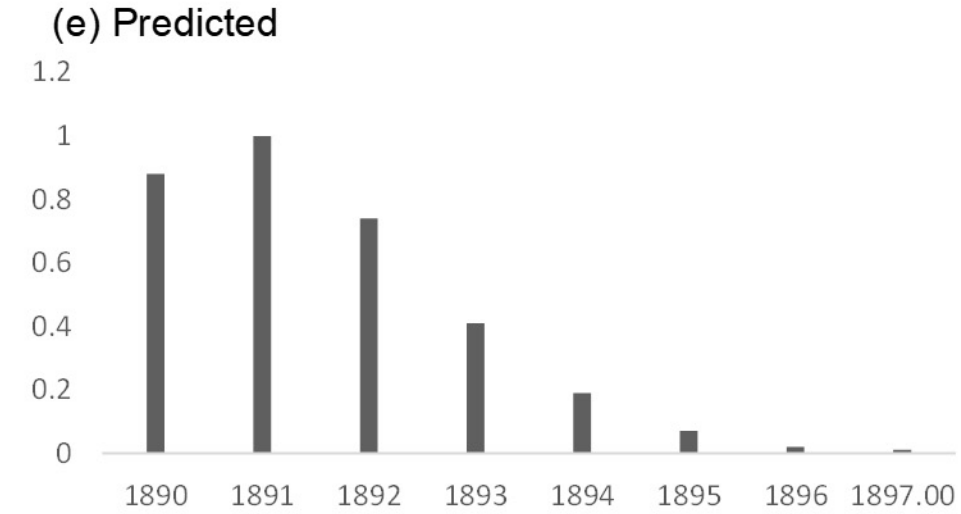

Figure S7. ESI-MS spectrum of compound **10** (CB-Tag). (a) Mass spectrum of compound **10**. ESI-MS m/z: [M - 2H$^+$]$^+$ calcd. for $C_{95}H_{148}N_9O_7S_2^{3+}$, 1592.11; found, 1592.1, [M + CF$_3$SO$^-$ - H$^+$]$^+$ calcd. for $C_{96}H_{149}F_3N_9O_{10}S_3^+$, 1742.06; found, 1742.0. [M + 2×CF$_3$SO$^-$]$^+$ calcd. for $C_{97}H_{150}F_6N_9O_{13}S_4^+$, 1892.02; found, 1891.9. Isotope pattern of single ion (OTf$^-$) adduct (b) observed, (c) predicted. Isotope pattern of double ion (2 × OTf$^-$) adduct (d) observed, (e) predicted.

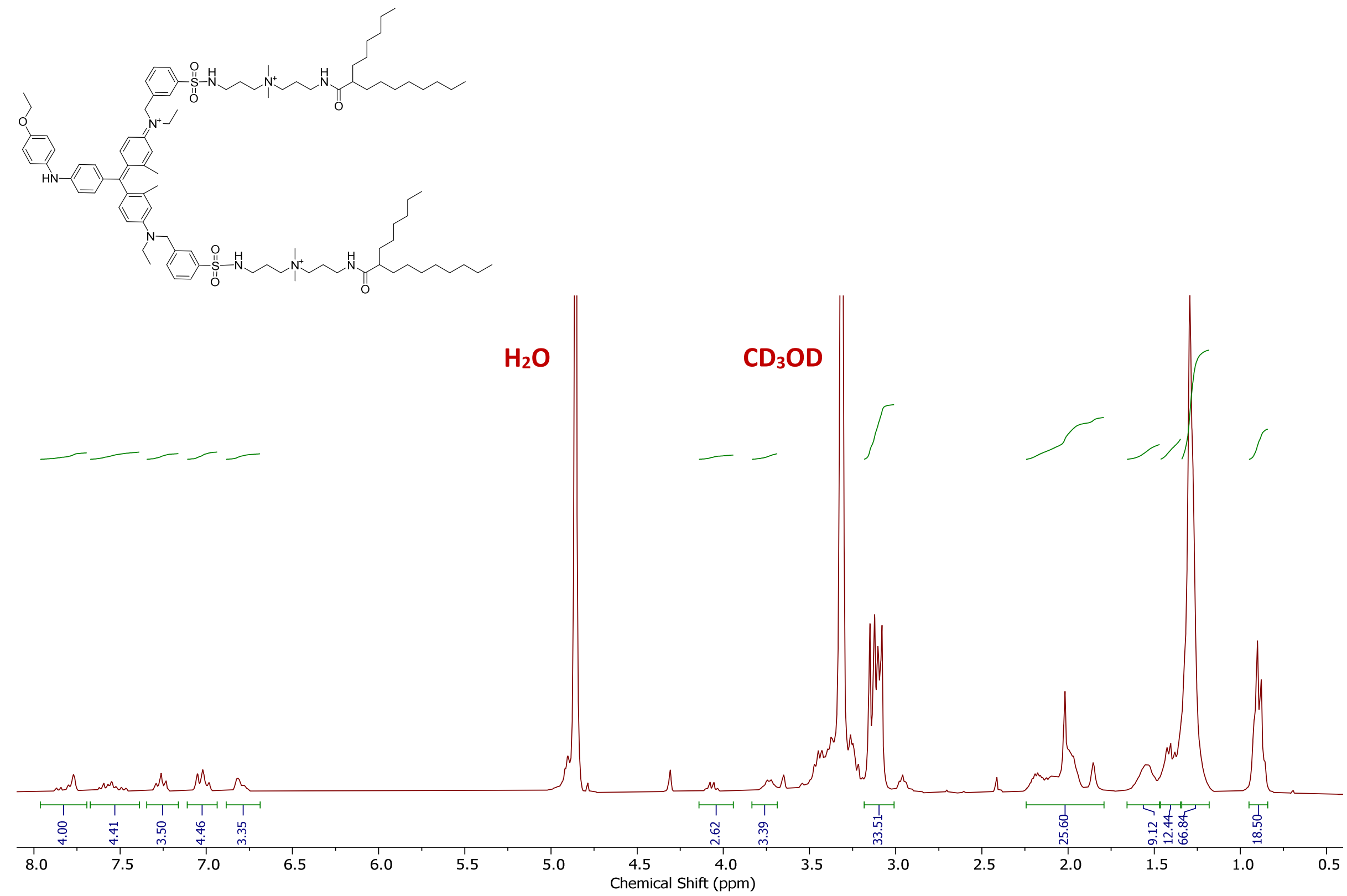

Figure S8. ^{1}H NMR spectrum of compound **10**. NMR was measured in CD_3OD.

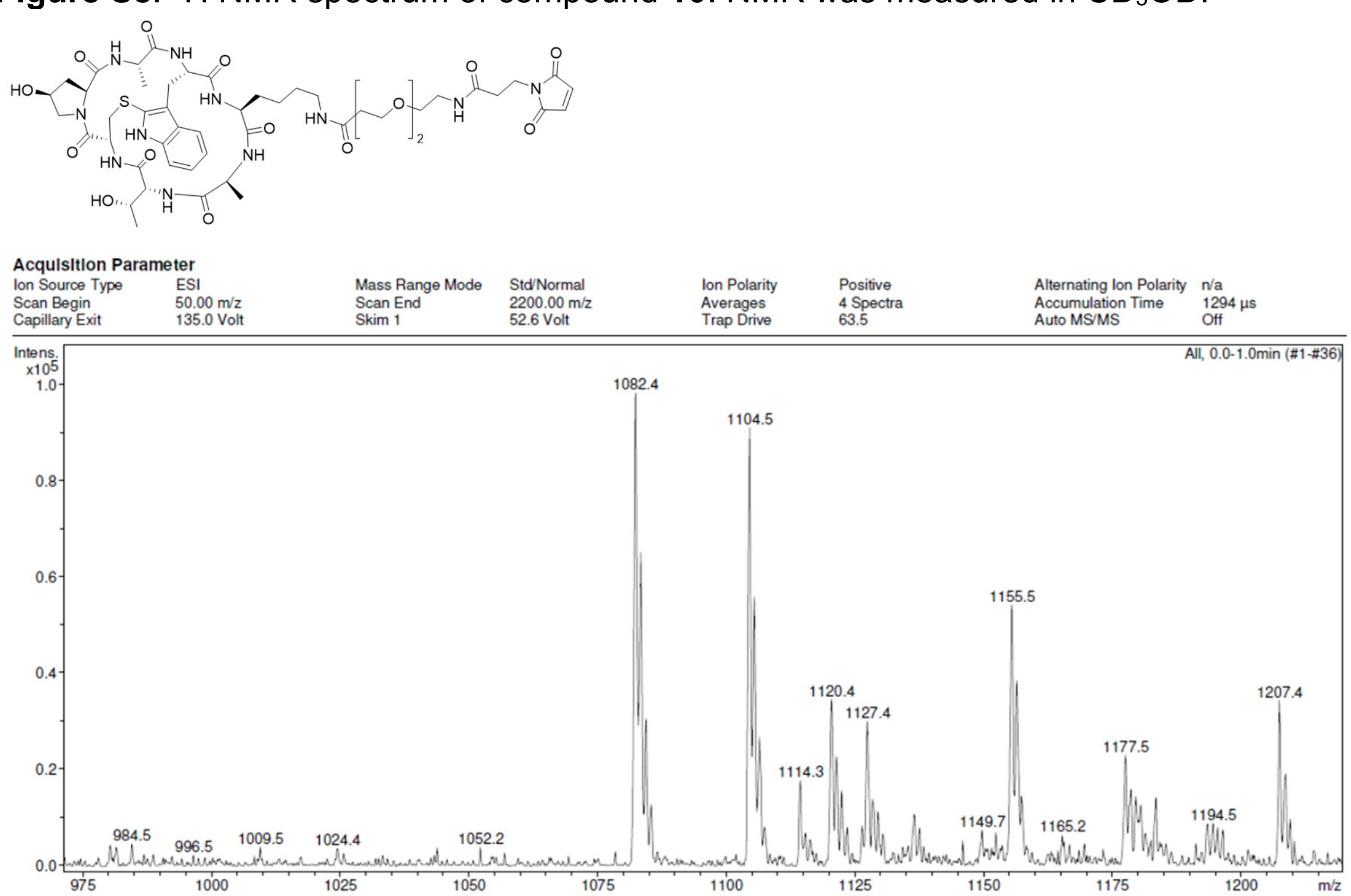

Figure S9. ESI-MS spectrum of Phalloidin-PEG2-Maleimide. ESI-MS m/z: $[M + H^{+}]^{+}$ calcd. for $C_{49}H_{68}N_{11}O_{15}S$, 1082.45; found, 1082.4, $[M + Na^{+}]^{+}$ calcd. for $C_{49}H_{67}N_{11}O_{15}SNa$, 1104.44; found, 1104.5.

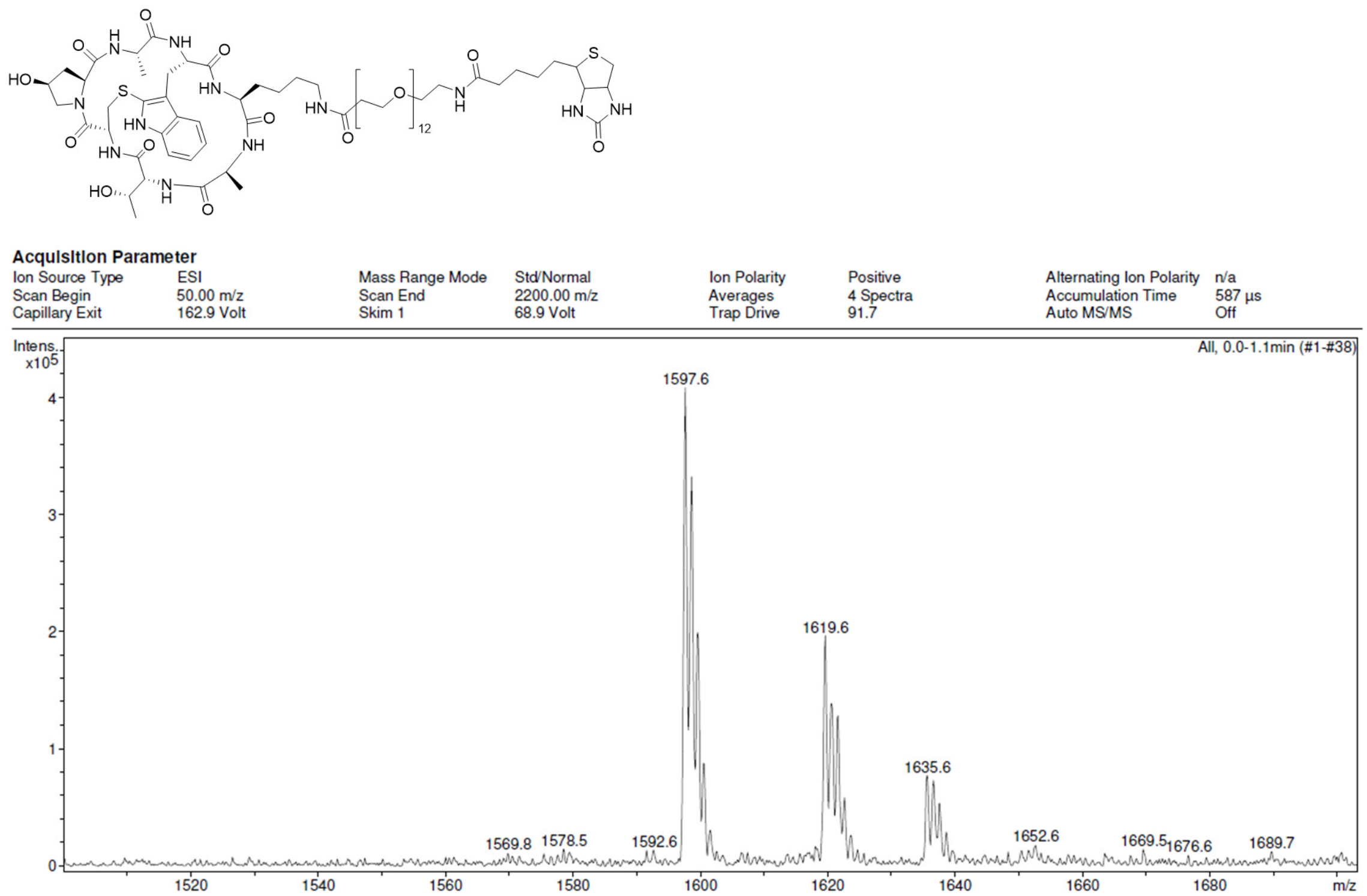

Figure S10. ESI-MS spectrum of Phalloidin-PEG12-Biotin. ESI-MS m/z: [M + H⁺]⁺ calcd. for $C_{72}H_{117}N_{12}O_{24}S_2$, 1597.77; found, 1597.6, [M + Na⁺]⁺ calcd. for $C_{72}H_{116}N_{12}O_{24}Na$, 1619.76; found, 1619.6.

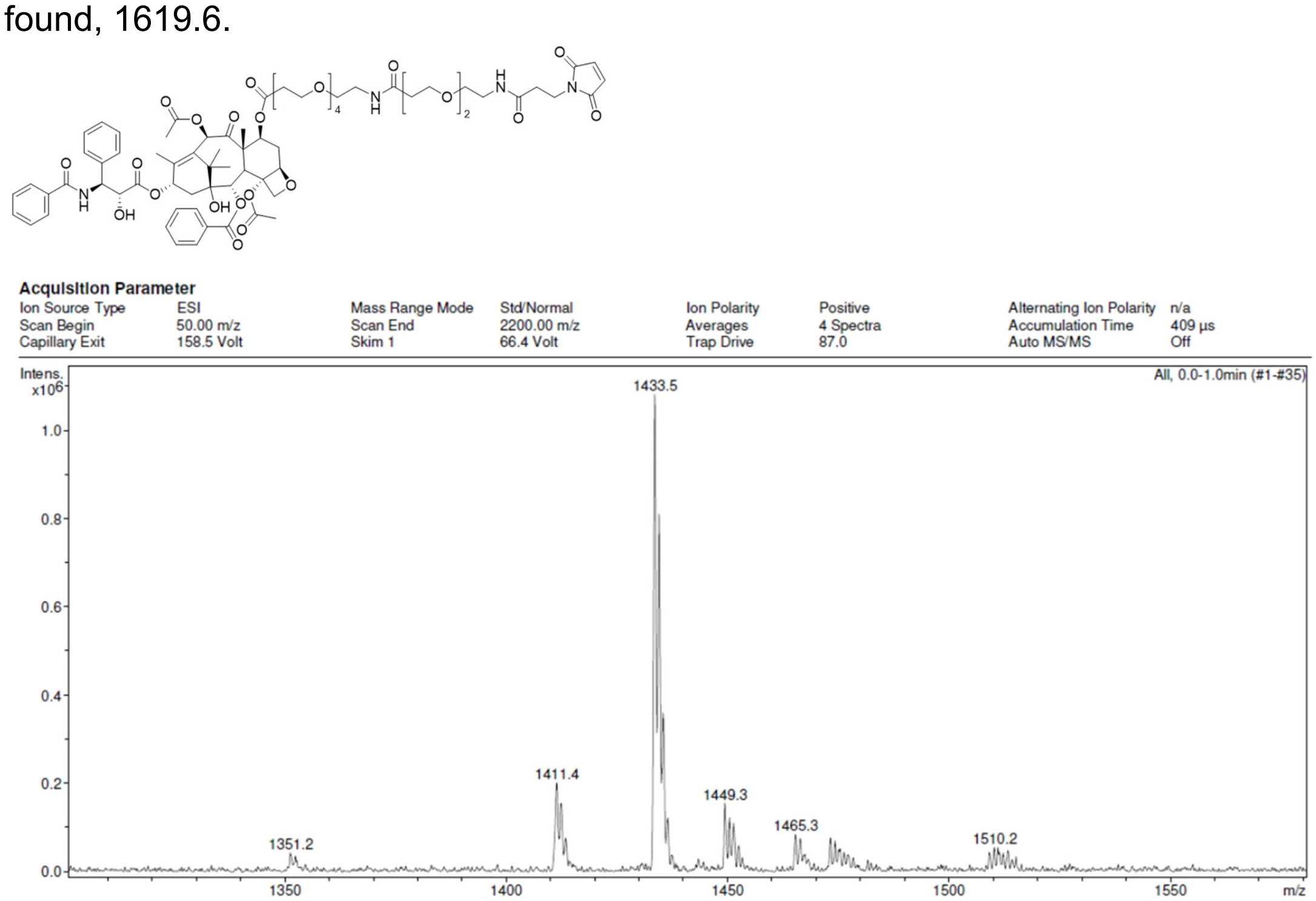

Figure S11. ESI-MS spectrum of PTX-PEG6-Maleimide. ESI-MS m/z: [M + H⁺]⁺ calcd. for $C_{72}H_{91}N_4O_{25}$, 1411.59; found, 1411.4, [M + Na⁺]⁺ calcd. for $C_{72}H_{90}N_4O_{25}Na$, 1433.58; found, 1433.5.

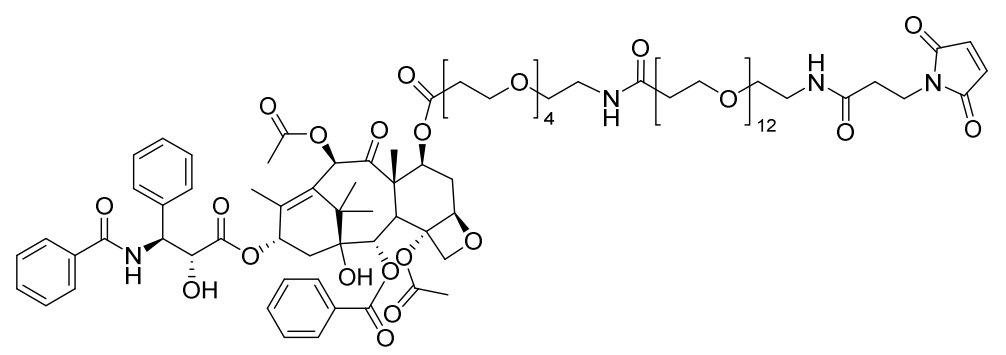

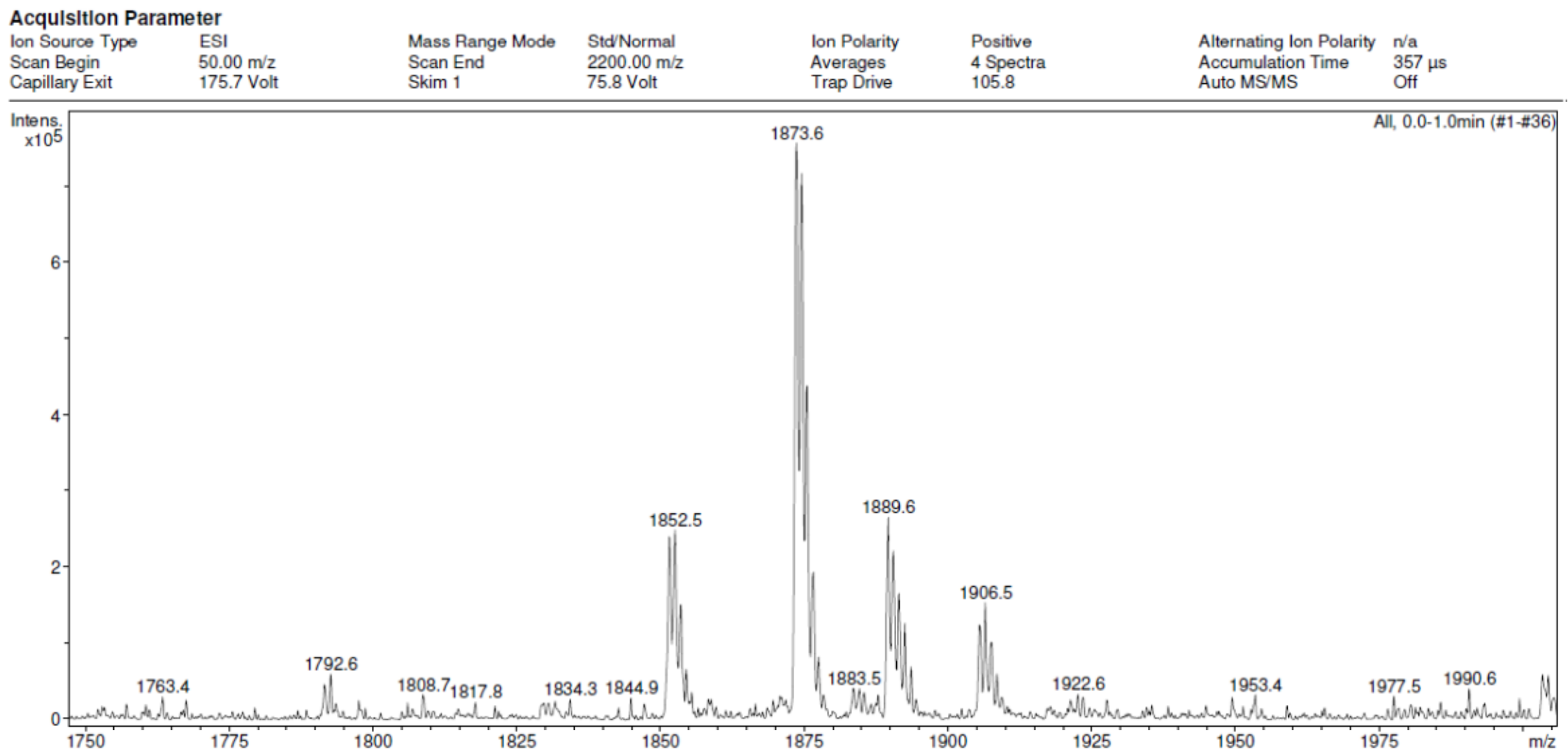

Figure S12. ESI-MS spectrum of PTX-PEG16-Maleimide. ESI-MS m/z: $[M + H^+]^+$ calcd. for : $C_{92}H_{131}N_4O_{35}$, 1852.85; found, 1852.5, $[M + Na^+]^+$ calcd. for : $C_{92}H_{130}N_4O_{35}Na$, 1873.84; found, 1873.6.

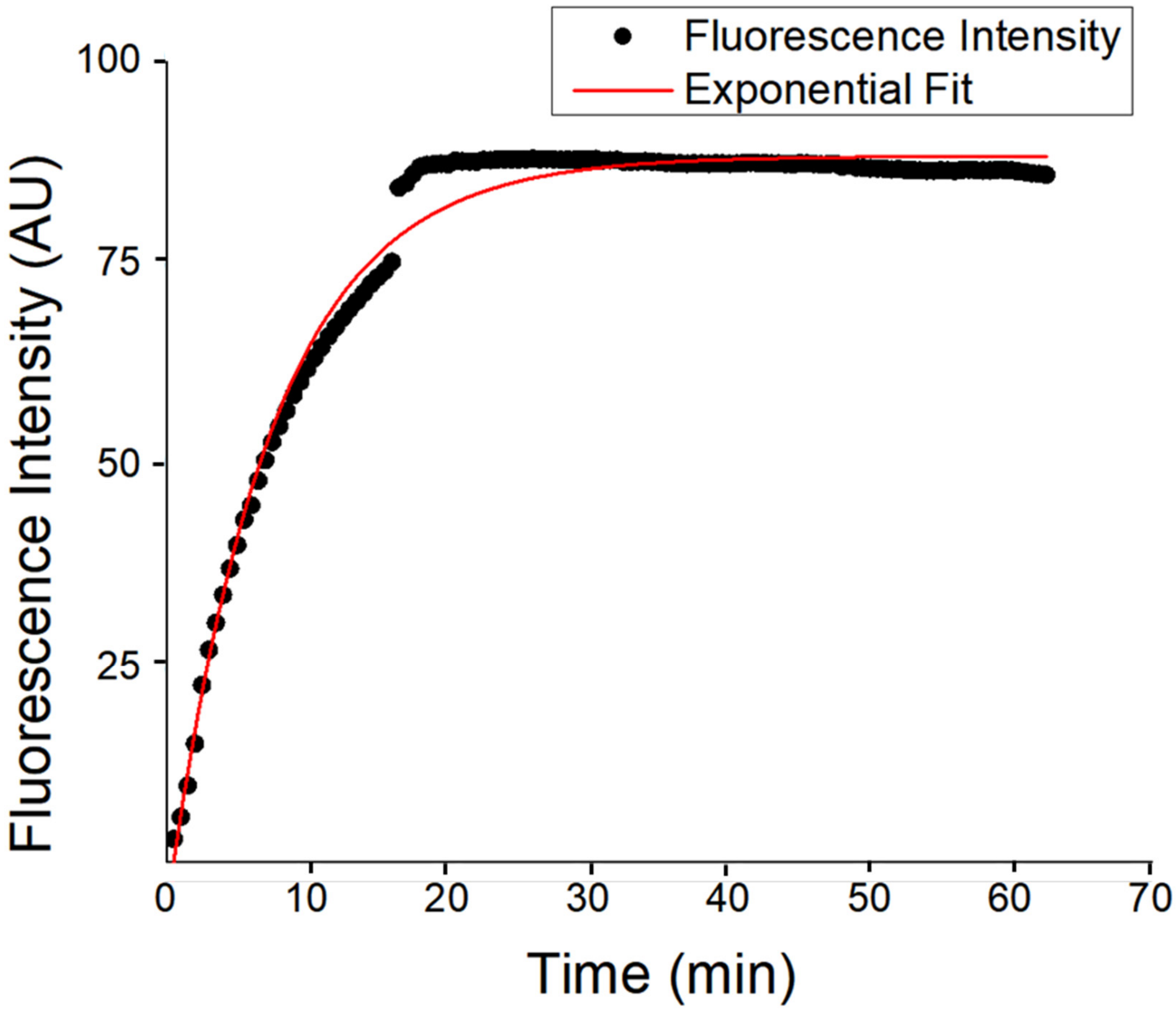

Figure S13. Fluorescence intensity change of the cell in Figure 3B as a function of time. Fluorescence intensity reaches to a plateau after ~20 min.

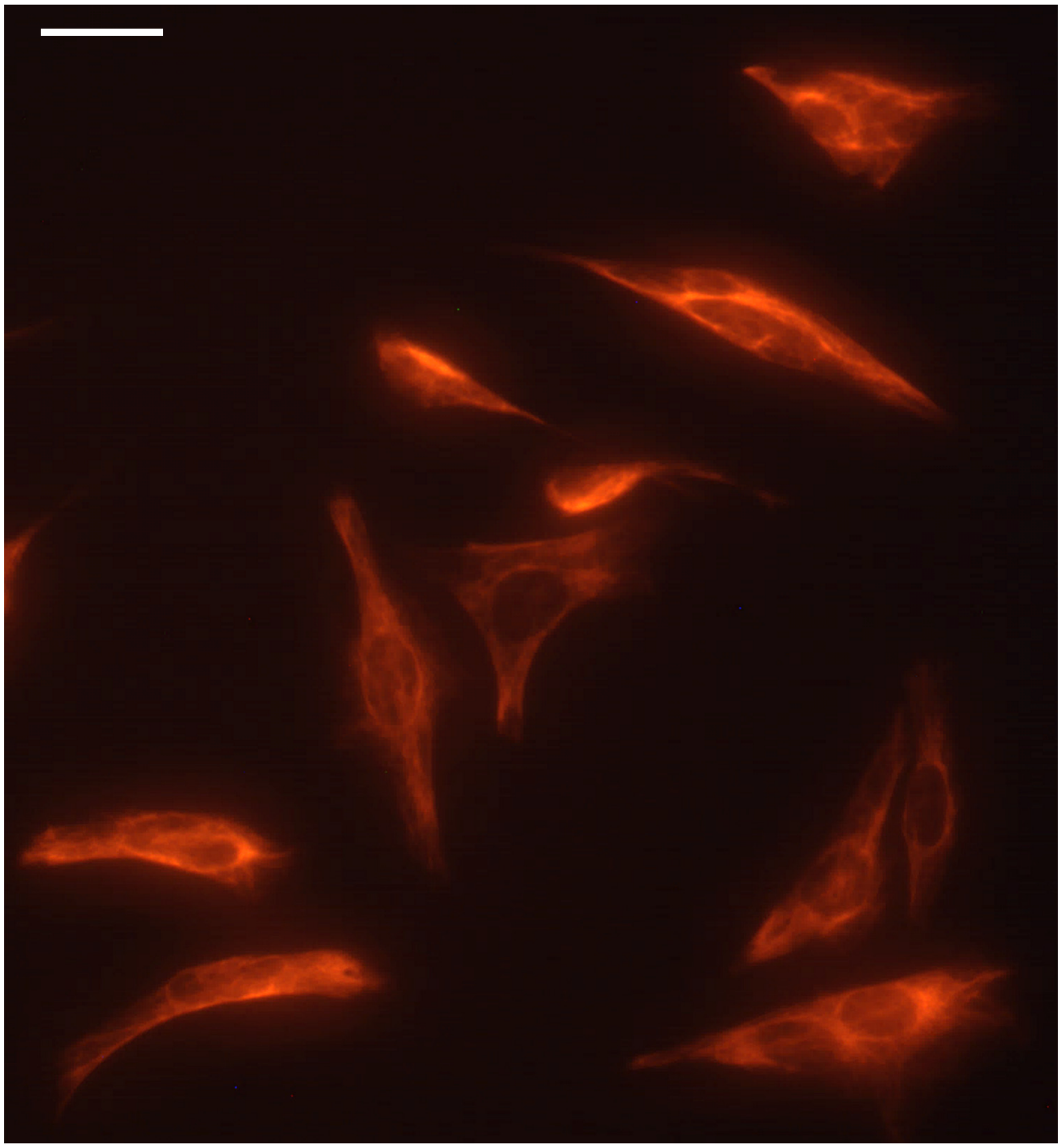

Figure S14. Representative image of fixed HeLa cells stained with anti-vimentin IgG-AF568. (HeLa cells were fixed with 4% paraformaldehyde for 10 min, permeabilized with 0.1% TX-100 for 10 min, and blocked with 1% BSA for 1 h at RT. The cells were labeled with anti-vimentin IgG-AF568 at 2 μg/mL in 0.1% BSA and incubated for 3 h at RT, and washed with PBS. Scale bar denotes 20 μm.

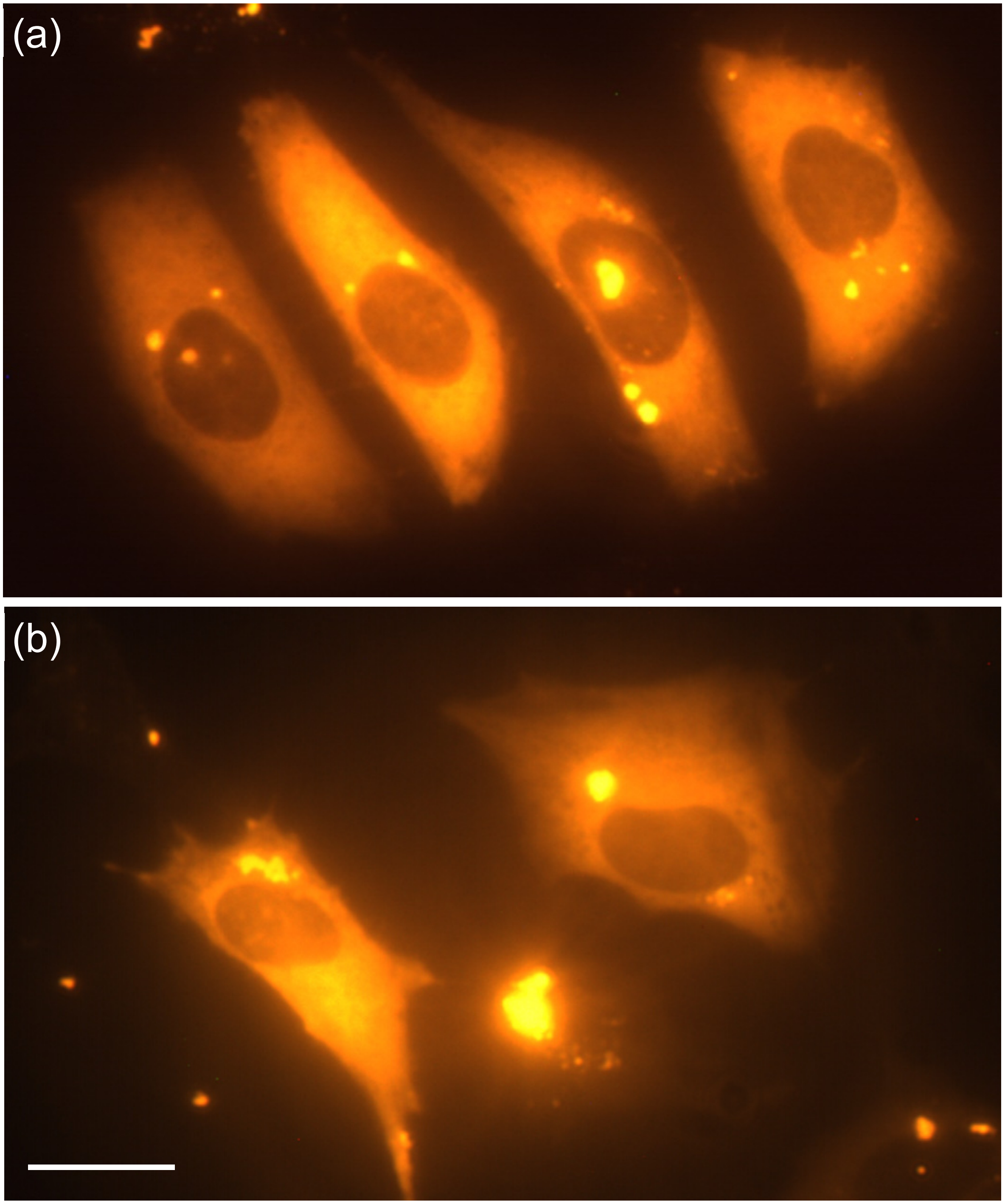

Figure S15. Representative fluorescence images of HeLa cells after intracellular delivery of (a) anti-ß-Tubulin IgG-AF555, or (b) anti-Actin IgG-AF555. [IgG-AF555]=50 nM, Tag ratio 1:10. Scale bar denotes 20 µm.

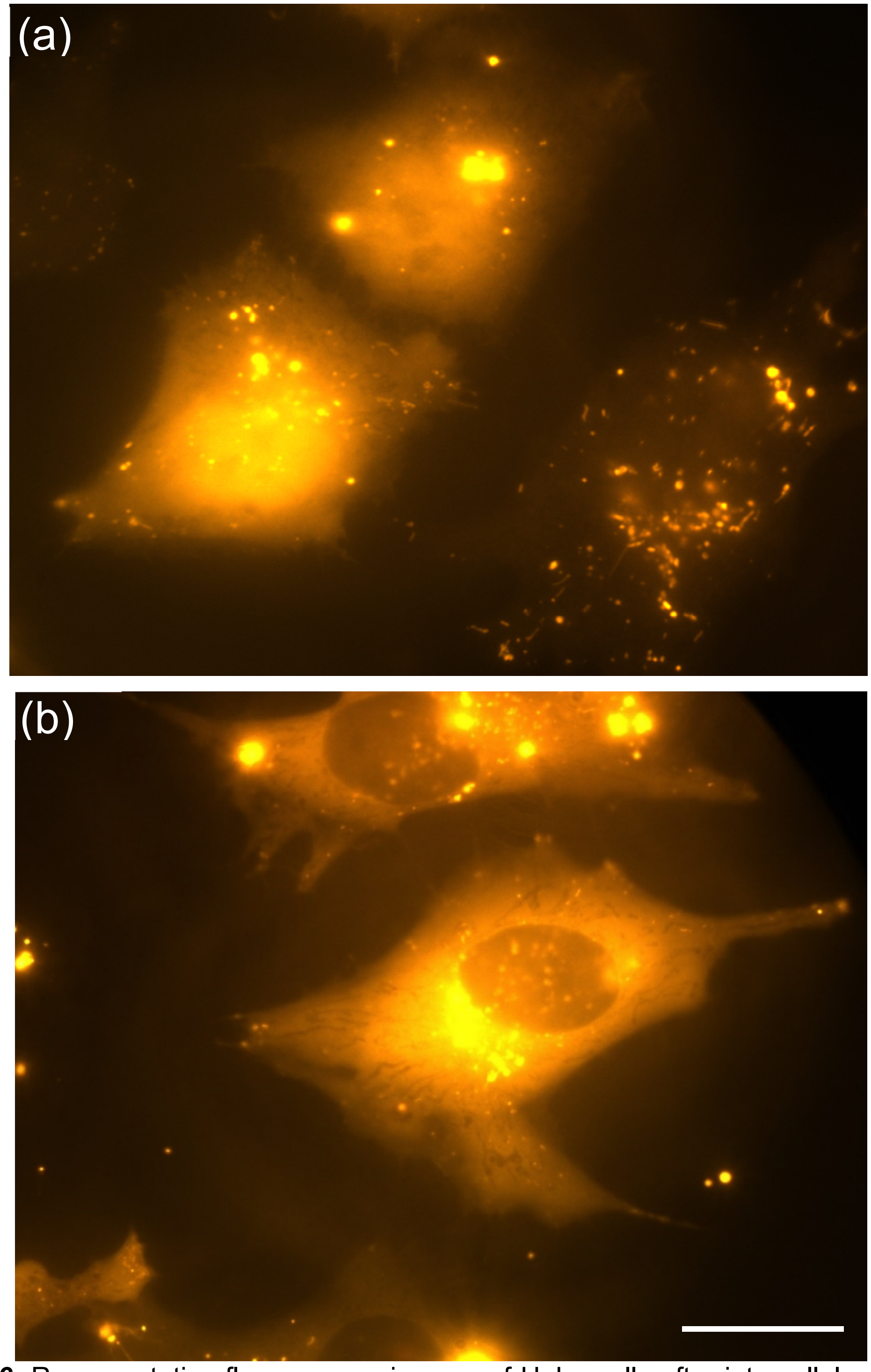

Figure S16. Representative fluorescence images of HeLa cells after intracellular co-delivery of Phalloidin-XX-Biotin (4X molar equivalent) with (a) Avidin-AF555. (100 nM, Tag ratio=1:2), or (b) Streptavidin-AF555. (100 nM, Tag ratio=1:2). Scale bar denotes 20 μm.

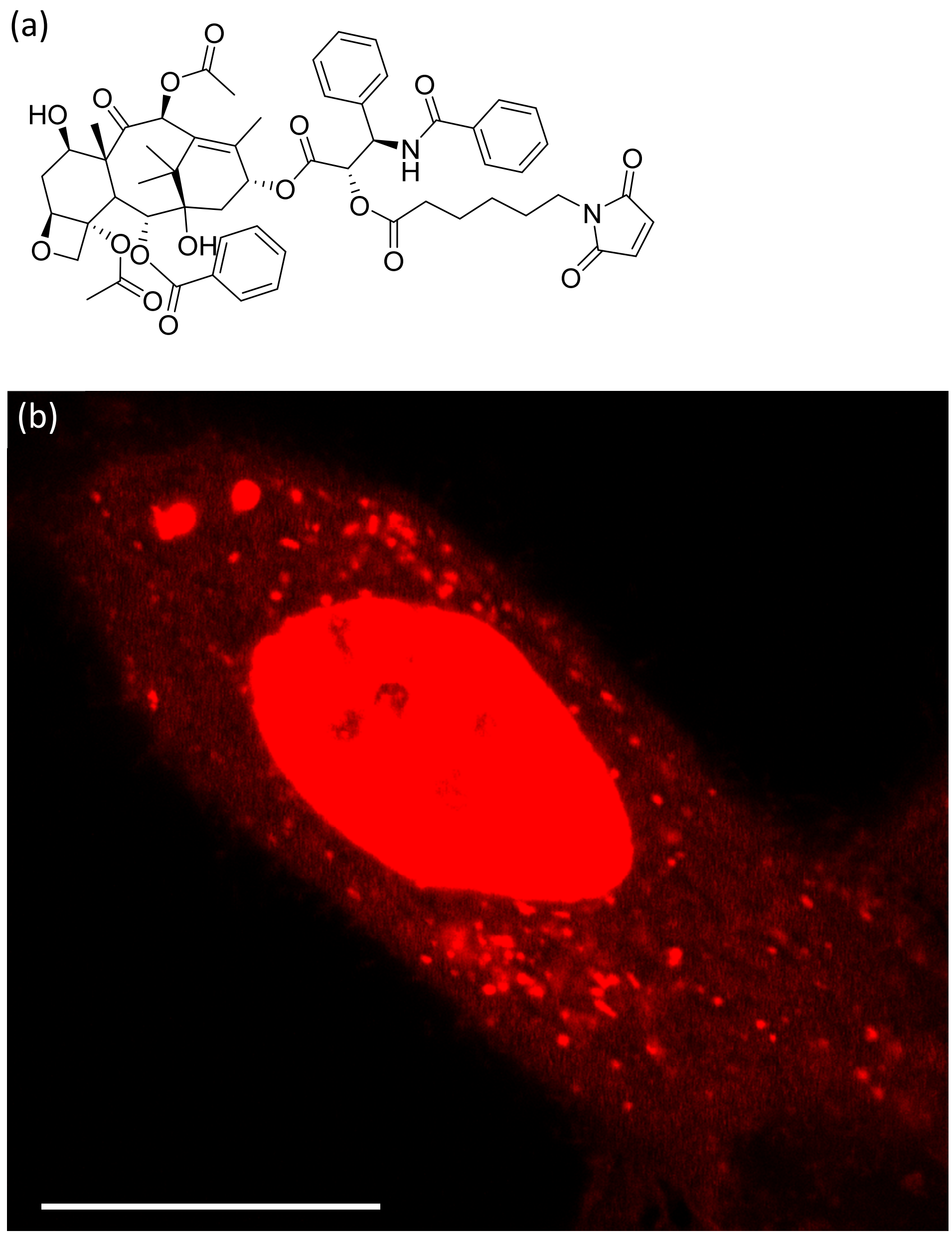

Figure S17. Intracellular delivery of PTX-Aprotinin-AF555. (a) Chemical structure of Paclitaxel conjugated with hexyl Maleimide at 2'-OH position. (b) Representative confocal images of HeLa cells after intracellular delivery of PTX-Aprotinin-AF555 conjugated at 2'-OH position. (50 nM, Tag ratio=1:2) Scale bar denotes 20 μm.

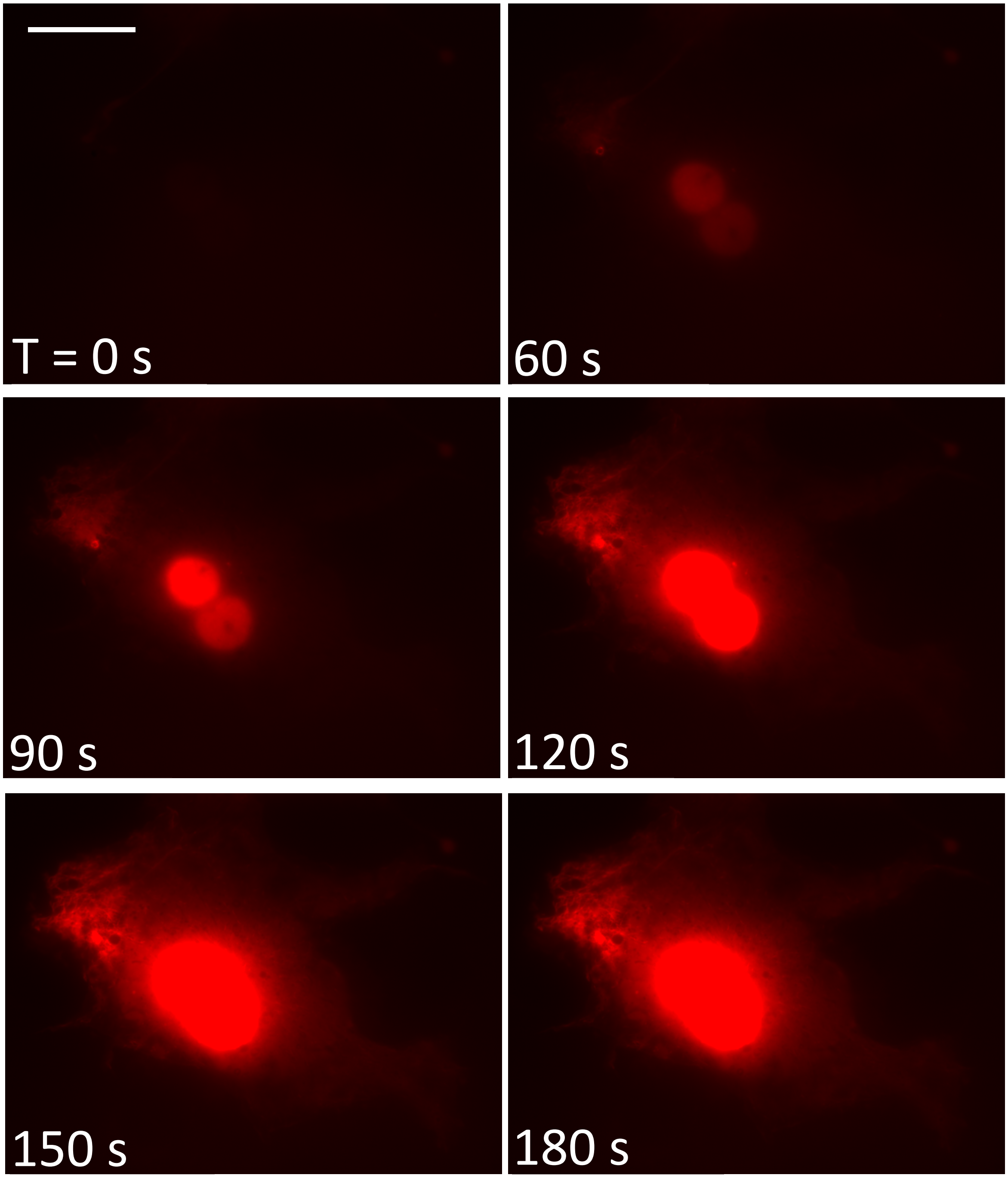

Figure S18. Zoomed-out view of **Figure 6d**. Scale bar denotes 20 μm.
